# *Prostanthera* (Lamiaceae) as a ‘Cradle of Incense’: Chemophenetics of Rare Essential Oils from Both New and Forgotten Australian ‘Mint Bush’ Species

**DOI:** 10.3390/plants9111570

**Published:** 2020-11-13

**Authors:** Nicholas J. Sadgrove, Guillermo F. Padilla-González, Ian R. H. Telford, Ben W. Greatrex, Graham L. Jones, Rose Andrew, Jeremy J. Bruhl, Moses K. Langat, Ingrid Melnikovova, Eloy Fernandez-Cusimamani

**Affiliations:** 1 Jodrell Science Laboratory, Royal Botanic Gardens Kew, Richmond TW9 3DS, UK; f.padilla@kew.org (G.F.P.-G.); m.langat@kew.org (M.K.L.); 2 Botany and N.C.W. Beadle Herbarium, School of Environmental and Rural Science, University of New England, Armidale, NSW 2351, Australia; itelford@une.edu.au (I.R.H.T.); jbruhl@une.edu.au (J.J.B.); 3 School of Science and Technology and School of Rural Medicine, University of New England, Armidale, NSW 2351, Australia; bgreatre@une.edu.au (B.W.G.); gjones2@une.edu.au (G.L.J.); 4 Ecosystem Management, School of Environmental and Rural Science, University of New England, Armidale, NSW 2351, Australia; randre20@une.edu.au; 5 Department of Crop Sciences and Agroforestry, Faculty of Tropical AgriSciences, Czech University of Life Sciences Prague, Kamýcká 129, 16500 Prague, Czech Republic; melnikovova@ftz.czu.cz

**Keywords:** chemophenetics, essential oils, taxonomy

## Abstract

The highly aromatic Australian mint bushes from the genus *Prostanthera* Labill. produce a high yield of essential oil on hydrodistillation. Together with its rich history, horticultural potential, iconic flowers, and aromatic leaves, it achieves high ornamental and culinary value. Species in the genus express highly diverse and chemically unique essential oils that demonstrate intra- and inter-specific patterns that have inspired taxonomic reinterpretation for over a hundred years. Previous studies have conveyed that phenoplastic expression of volatiles creates chemotypes within taxa, adding complexity to chemophenetic exploration. The current study chemically characterised essential oils from 64 highly aromatic specimens, representative of 25 taxa, giving yields as high as >2% g/g. The chemical profiles of essential oils are diverse, but generally include 1,8-cineole and signatory compounds such as sesquiterpene oxides, caryophyllene oxide, kessane and cis-dihydroagarofuran; sesquiterpene alcohols, globulol, epiglobulol, maaliol, prostantherol, spathulenol and ledol; and monoterpene derivatives of common scaffolds, borneol, bornyl acetate, bornanone, linalool and linalyl acetate. As in previous studies, analysis of chemical data confirms that the chemistry strongly agrees with taxonomic classifications. Importantly, as in classical taxonomy, the current chemical study complemented morphological analysis but conveys chemovariation, obscuring the taxonomic agreement. Nevertheless, variation within taxa may be due to environmental factors, meaning that cultivation of species in gardens will create different chemical profiles as compared to those published here.

## 1. Introduction

In 1978, the late Australian botanist, George William Francis Althofer (1903–1993), released his book ‘*Cradle of Incense: The story of Australian Prostanthera*’ [1]. Althofer procured a vast collection of species of *Prostanthera* Labill. for cultivation at Burrrendong Botanic Garden and Arboretum, which he founded outside of Wellington (NSW, Australia) in 1964. Through his collection, Althofer became aware of the true diversity within the genus and recognised new species, but some were never published, such as *Prostanthera* species Ulan (*P.* sp. Ulan; currently circumscribed as *P. rotundifolia* R.Br.).

The Royal Botanic Gardens Kew (London) describes the genus *Prostanthera* as having high horticultural value. Hence, in modern times, Australian home gardens are seeing a steady increase in species of *Prostanthera* under the common name ‘Australian mint bush’, which is partly due to the efforts of Althofer and modern indigenous plant nurseries. Species that are available commercially are often marketed as ‘culinary herbs for seasoning and flavouring’ [2]. This includes *P. rotundifolia* R.Br., and *P. incisa* R.Br., which add a eucalyptus flavour to jellies and chutneys. Some species are also known for medicinal effects in the treatment of topical afflictions, such as *P. rotundifolia*, which is made into ointments for sores and infections [3], or *P. striatiflora* F.Muell., which is used in fumigation modalities for lung afflictions [4,5,6].

**Figure 1:**
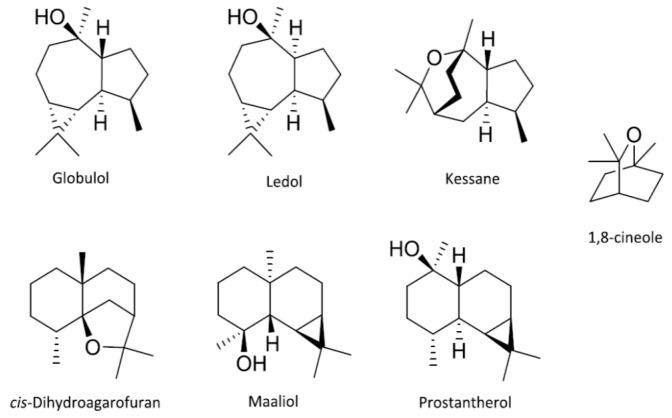
1,8-cineole and key sesquiterpenes in *Prostanthera*.

The essential oils from species in *Prostanthera* are generally rich in 1,8-cineole and key sesquiterpenes (Figure 1). However, taxa in the *P. lasianthos* Labill. heterogeneous species aggregate are atypical and were published earlier [7,8,9,10]. Over the course of multiple studies, it was realised that the two most prominent factors affecting the yield and chemical character of volatiles from *P. lasianthos* and closely allied species are (1) taxa and (2) soil or microclimate. It was hypothesised that drained soils influenced lower yields of volatiles that produced less 1,8-cineole and display a higher relative abundance of the other components, such as linalyl acetate, caryophyllene or β-selinene. However, even after acknowledging this phenotypic plasticity, a taxonomic agreement could also be demonstrated, giving intra-specific and inter-specific chemotypes [8].

In the *P. lasianthos* heterogeneous aggregate, chemical variability within a single taxon conformed to a continuum between two extreme chemical profiles, i.e., from 0 to >80% of 1,8-cineole, corroborating phenotypic plasticity. While this was evident from the study of *P.* sp. Wollomombi Gorge (J.B.Williams NE73839) [8], preliminary data on other taxa conveyed a general trend toward a similar conclusion. Hence, the chemical profile of volatile organic compounds can be diagnostic of single taxa, provided that the identities and patterns of phenoplastic volatile organic compounds within single taxa are understood. Examples of phenoplastic compounds are 1,8-cineole (*P. sp.* Wollomombi Gorge), α-pinene (*P. eungella* B.J.Conn and K.M.Proft), linalyl acetate (*P. sp.* Wollomombi Gorge), just to name a few [8].

There are currently 137 species of *Prostanthera* recognised on The Plant List [11]; however, the actual number of species is much higher, since there are many that are hitherto unrecognised. As demonstrated in previous studies, there are at least seven new taxa in the *P. lasianthos* heterogeneous aggregate [8] and more that are incorrectly circumscribed or affiliated with another taxon. For example, the Australasian Virtual Herbarium catalogues multiple *Prostanthera* with only phrase names [12]. Most of the taxa included in the current study are commonly identified incorrectly as *P. ovalifolia* R.Br. or *P. rotundifolia* and their correct names have remained unused and forgotten. Other taxa have been given only tentative names by affiliation but require a published morphological characterisation and incorporation into the taxonomic key.

Fortunately, the widespread inclusion of species of *Prostanthera* in the marketplace has aided in the conservation of rare and endangered genotypes, but it has also exacerbated the miscarriage of species identification. This confusion is echoed in the numbers of mis-determinations made on vouchers lodged at herbariums or in field surveys. Another layer of complexity is from the phenotypic plasticity of chemistry and morphology in relation to soil and climate. Thus, due to the waxing importance of the ‘mint bushes’ in horticulture and the challenges faced in taxonomy at large, a chemophenetic study is timely. The current study is focused on high essential oil yielding taxa, with other low yielding specimens included for comparison purposes. The main objective is to explore the phenetic relationships among taxa of *Prostanthera* using only the chemistry and yield of volatiles, giving comparison to morphology and taxa where appropriate, and to highlight the true diversity within the genus.

## 2. Materials and Methods

### 2.1. Specimen Collection

**Figure 2:**
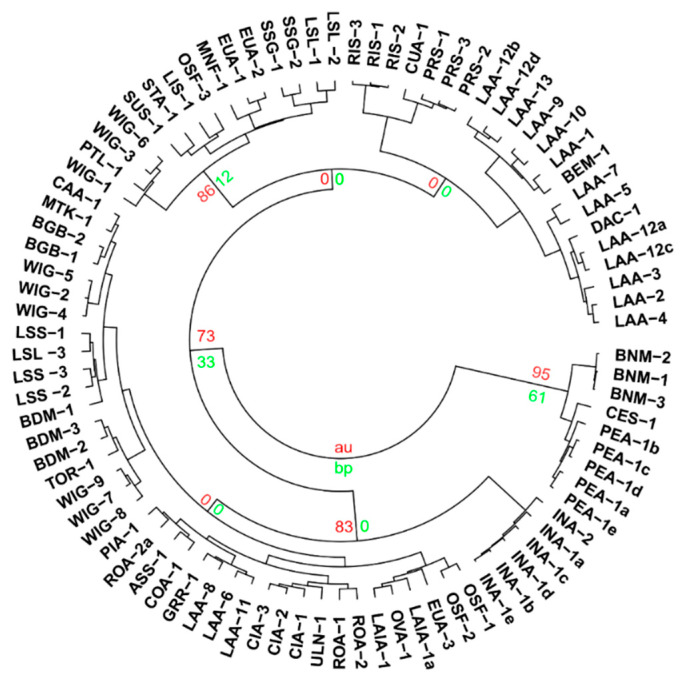
Dendrogram of all essential oil chemical data from the current study and published data [8,13]. BNM = *P.* sp. Barren Mountain, LAA = *P. lanceolata*, BEM = *P.* sp. Blue Mountains, CIA = *P. cineolifera*, ULN = *P.* sp. Ulan, COA = *P. cotinifolia*, OVA = *P. ovalifolia* (actual), ROA = *P. rotundifolia* (actual). CES = *P. centralis*, SUS = *P. suborbicularis*, STA = *P. striatiflora*, MNF = *P.* sp. Minyon Falls, OSF = *P.* sp. Olney State Forest, CUA = *P. cuneata*, PRS = *P. prunelloides,* LIS = *P. lithospermoides*, RIS = *P. ringens*, INA = *P. incisa*, CAA = *P. caerulea*, BGB = *P.* sp. Baking Board, TOR = *P.* sp. Thredbo River, GRR, *P.* sp. Gibraltar Range, ASS = *P. aspalathoides*, PIA = *P.* sp. Piliga, DAC = *P.* sp. Dandarha Creek. PEA = *P. petraea*. LSL = *P. lasianthos* subsp. *lasianthos*, LSS = *P. lasianthos* subsp. *suborbicularis*, EUA = *P. eungela*, BDM = *P.* sp. Bald Mountain, MTK = *P.* sp. Mt Kaputar, PTL = *P. sp.* Point Lookout, SSG = *P.* sp. Schofield’s Gap, WIG = *P.* sp. Wollomombi Gorge, and CES = *P. centralis*.

The locations of field specimens of all taxa and names are summarised in the Supplementary Files, Tables S1 and S2. Table S2 details the data that are published elsewhere but also included in the analysis that produced Figure 2. Vouchers of each accession were lodged at the N.C.W Beadle Herbarium at the University of New England, Armidale, NSW Australia. Data used from previous studies are available from the published literature [8,13,14].

### 2.2. Sample Preparation and Hydrodistillation

Essential oils were produced using hydrodistillation. Approximately 600 g of fresh leaf was removed from the twig then cut into 0.5 mm fragments and placed into a 5 L round bottom flask with 2.5 L of deionised distilled water (ddH_2_O). Leaves were heated for 3–4 h using continuous cohobation of hydrosol. Afterwards the steam/oil mix was collected from the 1000 mL separating funnel after separating from the hydrosol. Essential oils were stored away from light at 4 °C until used.

The condenser was rinsed with hexane after all distillations to collected sesquiterpenes that had solidified against the glass cooling coils. The total yields reported in Table 1, Table 2, Table 3, Table 4 and Table 5 include the recovered sesquiterpenes.

### 2.3. Chromatography Methods and Compound Identification (GC–MS and NMR Analysis)

Prior to GC–MS analysis the essential oils were dried to remove hydrosol emulsions using anhydrous sodium sulphate (Na_2_SO_4_) powder (0.5 g in 10 mL essential oil) for at least 24 h. Afterwards, essential oils were dissolved in dichloromethane (DCM) at a ratio of 1:1000.

Analyses were performed using a HP 6890 gas chromatograph coupled with a HP 5973 mass spectrometer detector. An autosampler unit (HP 7673–100 positions) was used to perform the 1 μL injections. Separation was accomplished with a HP-5MS column (30 m by 0.25 mm, i.d., 0.25 μm phase thickness). Operating conditions were as follows: injector: split ratio 25:1; temperature: 250 °C; carrier gas: helium, 1.0 mL/min, constant flow; column temperature, 60 °C (no hold), 5 °C per minute then @ 280 °C hold for 4 min. MS was acquired at 70 eV using a mass scan range of 45–300 *m*/*z*. Quantification was based on the peak areas on the chromatogram generated by MS data (total ion chromatogram). Integration parameters were set to exclude peak areas less than 0.5%.

Most identification of essential oil components was performed by comparison of mass spectra with the NIST electronic library database [15] and confirmed using calculated retention indices relative to n-alkanes, then compared to published values. A second library in the form of a book by Adams [16] was used to resolve any further discrepancies with identification. Most samples were injected once only, accept in cases of unusual or unexpected chemical profiles. These samples with repeats include the ROA replicates, the LAIA repeats, OVA, BEM, COA, CAA and ULN. Some of the components were identified by isolation (10% ethyl acetate, 90% hexane, normal phase column chromatography using silica gel) and comparison of respective ^13^C NMR spectra to published values, using a 500 MHz Bruker Avance (Germany) spectrometer in *d*-chloroform. These were; prostantherol, ledol, cis-dihydroagarofuran, maaliol, globulol, epiglobulol and kessane, which were also authenticated in previous studies by matching ^13^C NMR spectra to published values [17,18,19,20,21]. A snapshot of the ^13^C NMR spectrum for each of these sesquiterpenes is provided as Figures S1–S8 in Supplementary Files.

### 2.4. Multivariate Analysis

Percentage compositions of the essential oil components were used as input data to perform a hierarchical clustering analysis (HCAbp) with bootstrap resampling in the software R 3.4.1 (R Foundation for Statistical Computing, Vienna, Austria), using the pvclust [22] package, the ward’s clustering algorithm and Euclidean distance. Prior to multivariate analyses, raw data were scaled by the arcsine method in accordance with previous reports for data expressed as percentages [23,24]. Two types of support values were plotted in the HCAbp using 10,000 replicates: traditional bootstrapping (bp) and approximately unbiased (au) *p*-values.

A principal component analysis (PCA) was performed using the prcomp function and the factoextra package in the software R. Prior to PCA, raw data were scaled by the arcsine method in accordance with previous reports for data expressed as percentages [23,24].

## 3. Results and Discussion

### 3.1. The History of Essential Oil Studies in Prostanthera

In 1980, the late Erich V. Lassak (1934–2015) published his work on the chemical character of the essential oils from over 40 of those species in Althofer’s living collection at Burrrendong Botanic Garden and Arboretum [25]. Lassak observed that the taxonomic classifications of *Prostanthera*, as proposed by Bentham, correlated significantly to essential oil yield and chemistry. This observation was corroborated by Barry Conn when he distilled 44 specimens of *P. aspalathoides* A.Cunn. ex Benth. ranging from South Australia into Victoria and New South Wales, as a follow up to Lassak’s work [25,26]. Conn’s efforts convey that geography is a factor in determining the chemical character of that species [27].

Earlier studies on the chemistry of *Prostanthera* need to be revisited with consideration to the numerous unidentified components, and the new taxonomic insights that have been realised over the last 40 or so years [27,28,29]. The earliest chemical study published on a species of *Prostanthera* was by R.T. Baker and H.G. Smith in 1912 [30]. They chemically characterised an essential oil from a new species which they named *P. cineolifera* R.T.Baker and H.G.Sm in the same study. In their paper, they mention that essential oils from *Prostanthera* were of interest to Joseph Bosisto (1827–1898), a chemist and politician who founded the country’s biggest Eucalyptus oil company “Bosisto’s”. Allegedly in 1862 Bosisto distilled leaves from *P. lasianthos* and *P. rotundifolia* but lost interest in the former due to a low yield, and the latter (0.75% yield *w*/*w* fresh leaves) due to a strong similarity to Eucalyptus oil.

Another prominent chemist who worked on Australian essential oils was Arthur de Ramon Penfold (1890–1980) [31,32]. Penfold was the protégé of H.G. Smith at the Museum of Applied Arts and Sciences, Sydney, before taking over as director in 1927. Penfold distilled some species of *Prostanthera* from the Hunter Valley, which he collected from Putty and Mt Danger (Sandy Hollow, NSW). His work was later published by his own protégé R.O. Hellyer [33], but unfortunately there was some confusion in the labelling of Penfold’s samples, causing Hellyer to report the chemistry of the wrong species, which will be corrected in this report.

In the time since Lassak published his paper on *Prostanthera* [25], many of the unknown sesquiterpenes have been assigned. Nearly 30 years ago, Southwell and Tucker [17] identified kessane and assigned *Z*-dihydroagarofuran (Figure 1) by spectroscopic methods from essential oil of *P*. sp. aff. *ovalifolia* (*P. lanceolata* Domin.), then prostantherol was assigned a year later after it was isolated from the two species *P.* sp. aff. *melissifolia* and *P. rotundifolia,* growing at the Royal Botanic Gardens in Edinburgh, Scotland [18]. Prostantherol is now known as the main component of *P. centralis* B.J.Conn. essential oil [13].

### 3.2. The Multivariate Analysis

The dendrogram provided as Figure 2 conveys a high degree of taxonomic agreement, but it is evident that chemical variability within taxa is sometimes greater than variability across taxa, creating overlap. The chemotypes and plasticity in the *P. lasianthos* heterogeneous aggregate were explained in a previous study [8], which are thought to relate to soil type and/or drainage vs. moisture retention. Similar plasticity is evident for the specimens of the current study.

**Figure 3:**
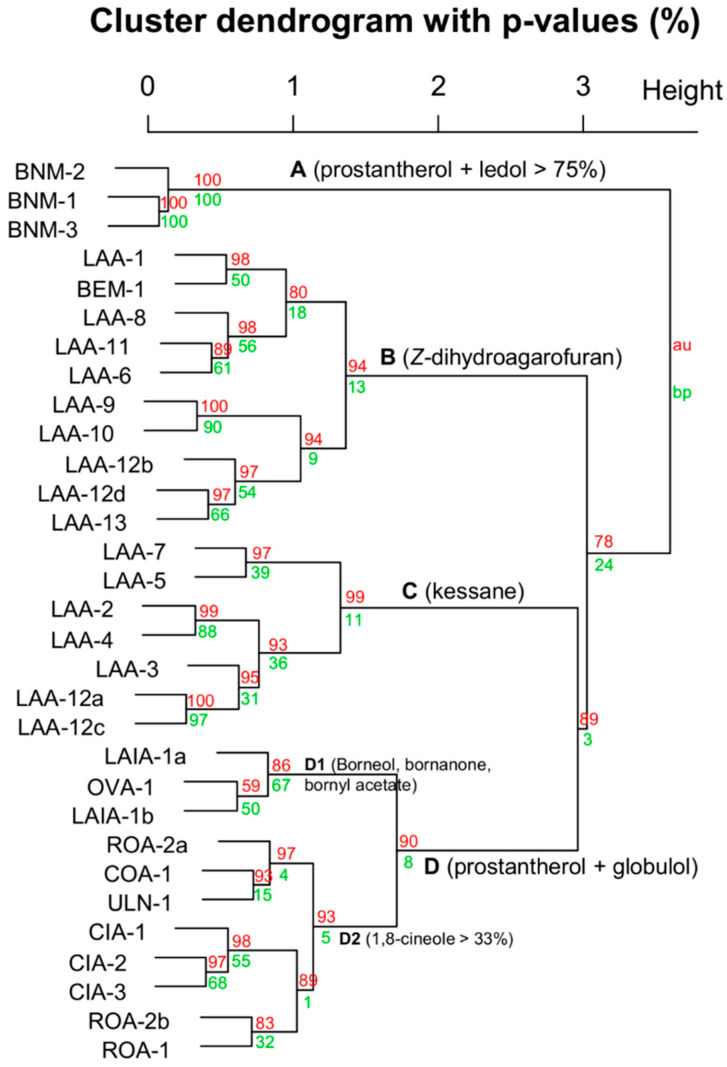
Dendrogram of chemical data of essential oils from species affiliated with *P. ovalifolia* and *P. rotundifolia*. BNM = *P. sp.* Barren Mountain, LAA = *P. lanceolata*, BEM = *P. sp.* Blue Mountains, CIA = *P. cineolifera*, ULN = *P. sp.* Ulan, COA = *P. cotinifolia*, OVA = *P. ovalifolia* (actual), and ROA = *P. rotundifolia* (actual).

**Figure 4:**
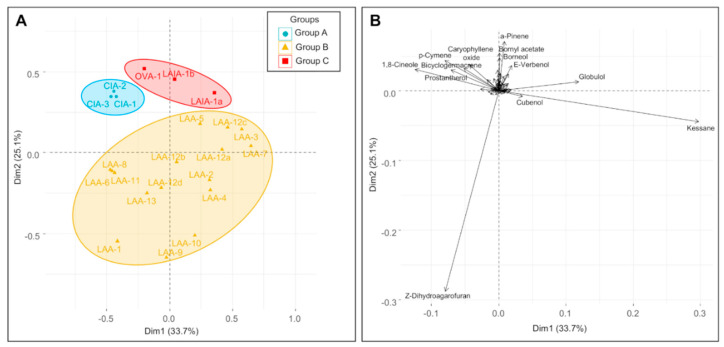
PCA scores plot (**A**) showing the four species most closely affiliated with *P. ovalifolia*, and loadings plot (**B**) showing the contribution of individual essential oil components to the segregation. LAA = *P. lanceolata*, CIA = *P. cineolifera*, OVA = *P. ovalifolia* (actual), and LAIA = *P. latifolia*.

**Figure 5:**
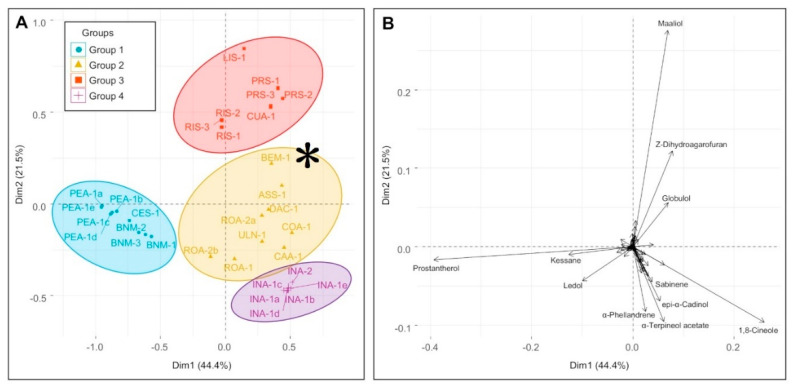
PCA scores plot (**A**) showing the four groupings of species outside the *P. ovalifolia* complex, and loadings plot (**B**) showing the contribution of individual essential oil components to the segregation. Group 1: PEA = *P. petraea*, BNM = *P.* sp. Barren Mountain, and CES = *P. centralis*. Group 2: BEM = *P.* sp. Blue Mountains, ASS = *P. aspalathoides*, DAC = *P*. sp. Dandarha Creek, ROA = *P. rotundifolia* (actual), ULN = *P.* sp. Ulan, COA = *P. cotinifolia*, and CAA = *P. caerulea*. Group 3: LIS = *P. lithospermoides*, RIS = *P. ringens*, PRS = *P. prunelloides*, and CUA = *P. cuneata*. Group 4: INA = *P. incisa*.

**Figure 6:**
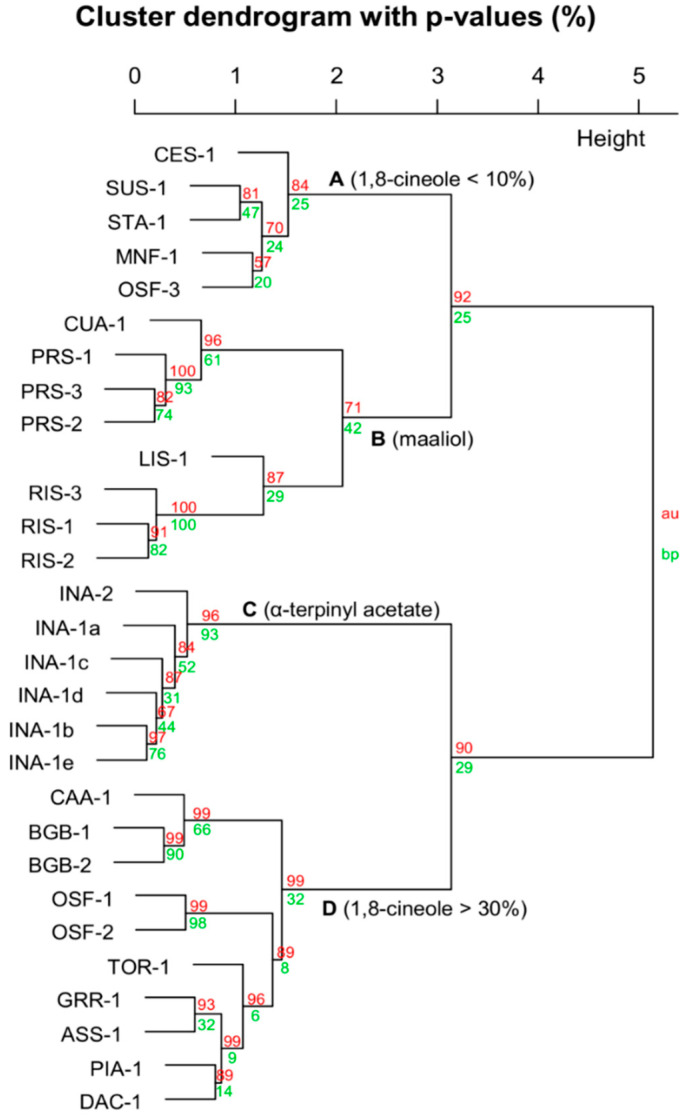
Dendrogram of chemical data from miscellaneous taxa in *Prostanthera*, not part of the *P. ovalifolia* or *P. rotundiolia* group. CES = *P. centralis*, SUS = *P. suborbicularis*, STA = *P. striatiflora*, MNF = *P.* sp. Minyon Falls, OSF = *P.* sp. Olney State Forest, CUA = *P. cuneata*, PRS = *P. prunelloides,* LIS = *P. lithospermoides*, RIS = *P. ringens*, INA = *P. incisa*, CAA = *P. caerulea*, BGB = *P.* sp. Baking Board, TOR = *P.* sp. Thredbo River, GRR, *P. sp.* Gibraltar Range, ASS = *P. aspalathoides*, PIA = *P.* sp. Piliga, and DAC = *P*. sp. Dandarha Creek.

Inevitably, taxonomic agreement to chemical datasets improves as the species diversity of the dataset is reduced. This is evident in the analysis in Figure 3, Figure 4, Figure 5 and Figure 6. Figure 3 includes all taxa with close affiliation to *P. ovalifolia* and *P. rotundifolia*, which are maaliol deficient high essential oil yielding specimens from section *Prostanthera*. Figure 4 gives a narrower analysis of species more closely related to *P. ovalifolia* using PCA. Figure 5 includes maaliol-rich species and the other taxa of the current study (excluding the data published elsewhere). In Figure 6, the maaliol-rich species are also analysed in PCA alongside the prostantherol-rich species and the *P. rotundifolia* complex.

### 3.3. Taxa Affiliated with Prostanthera Ovalifolia

*Prostanthera lanceolata* was first named in 1928 (Domin. Bibliotheca Botanica. Kassel: 89: 568, 1928), nearly 100 years ago, but the species has been continuously misidentified as *P. ovalifolia*. As far as is known its distribution spans from central coast NSW, inland toward the higher altitudes of the New England region, and northwards to the coastal mountains comprising the eastern side of the NSW-Qld border; going from arid sub-zero winter climates (New England NP) to warmer humid climates, respectively. The taxonomic confusion and continuous mis-determinations may be related to the high morphological variability from juvenile to adult specimens, where the strongly incised margins of juvenile leaves can be preserved into maturity, due to the process of stunting growth caused by rocky cliffs with poor rooting support.

Like other species in *Prostanthera* section *Prostanthera* [29], the highly aromatic foliage of *P. lanceolata* (LAA) expresses a significant amount of essential oil, with yields ranging from 0.5 to 2.0% g/g fresh leaves (Table 1). This species was the most represented out of the species of the current study, which was sampled 13 times. With four replicates of LAA-12, this gives 16 total specimens with characterised essential oils (Table 1). Unsurprisingly, even with multiple replicates from the one location (LAA-12: New England NP) a degree of chemical variation is evident between individuals. As previously mentioned, the intra-specific chemical variability within *P. lanceolata* is characterised by the profiles of kessane and *Z*-dihydroagarofuran (Figure 2) corroborating observations made in an earlier study of 43 specimens [17].

By examining the comparison of closely allied species in Figure 3, a distinct splitting of *P. lanceolata* Domin is evident. One group recognises kessane as the dominant sesquiterpene oxide and globulol (branch C; Figure 3) as the dominant sesquiterpene alcohol. The other group is the converse of this (branch B; Figure 3). In this regard, the phrase named *P. sp.* Blue Mountains (*P.* sp. aff. *rotundifolia*) was placed in the high *Z*-dihydroagarofuran branch (Figure 3).

The two sesquiterpene oxides kessane and *Z*-dihydroagarofuran were distilled from *P. lanceolata* and chemically assigned in the 1990s, but the species was identified as affiliated with *P. ovalifolia* and determined as *P.* sp. aff. *ovalifolia* [17]. Those authors observed the same variation as in the current study and proposed that different daytime temperatures may be responsible. With a greater geographic range of sampling in the current study, it is clear that geography or climate may be significant as proposed previously.

In the first instance, the most southernly specimens express lower relative amounts of the two oxide sesquiterpenes, kessane and *Z*-dihydroagarofuran. Southwell and Tucker proposed a hypothesis that the higher day time temperatures of the northern specimens favour the parallel twist conformation of *Z*-dihydroagarofuran in the course of its biosynthesis [17]. They also observed differences in the expression of sesquiterpene alcohols. Our observation is that hydronapthalenic *Z*-dihydroagarofuran and hydroazulenic kessane possibly modulate between hydronapthalenic prostantherol and hydroazulenic globulol, respectively (Figure 1). However, the exact reason for this is not immediately obvious. Nevertheless, since prostantherol was only described [18] a year after Southwell and Tucker’s study [17] it is possible that they overlooked this.

A comparison of the chemical character of *P. lanceolata* essential oil with that of the actual *P. ovalifolia* (OVA-1) conveys a noteworthy difference. Surprisingly, *P. ovalifolia* expresses an essential oil that is moderately sesquiterpene deficient (Table 2). Furthermore, the presence of borneol, bornanone (camphor) and bornyl acetate makes it relatively unique, comparable only to *P. latifolia* (LAIA-1a and -1b), which is another of the forgotten species. The convergence of their chemical profiles is evident in the dendrograms of Figure 2 and Figure 3. The earlier botanist George Bentham (1800–1884) regarded *P. latifolia* (Benth.) Domin as a variety of *P. ovalifolia* (i.e., *P. ovalifolia* var. *latifolia* Benth.) but its status at species rank is obvious in the light of modern taxonomy. Nevertheless, the close chemical relationship echoes Bentham’s observation of a close morphological relationship.

Initially, it was a challenge for us to identify *P. latifolia* because there were two similar species within proximity to its type locality. However, after chemical characterization, we were compelled to make a closer examination of the morphology and realised that one of the taxa constitutes a new species, which we have ascribed the phrase name *P.* sp. Barren Mountain (BNM-1, -2 and -3). This latter taxon produced an essential oil comprised by 65–80% of prostantherol + ledol, which is very similar to that produced by *P. petraea* B.J.Conn. (Table 5). Alternatively, *P. latifolia* expresses an essential oil that includes borneol, bornanone (camphor) and bornyl acetate as previously mentioned.

The chemical relationships of taxa in the *P. ovalifolia* complex was re-examined using principle component analysis (PCA) (Figure 4), focusing on the four species. PCA clearly demonstrated a difference between *P. cineolifera* and *P. ovalifolia*, but also reiterates the close chemical relatedness of *P. ovalifolia* and *P. latifolia*. The loadings plot (Figure 4B) conveys that the strongest chemical discriminators are *Z-*dihydroagarofuran and kessane as previously discussed.

The etymology of *P. cineolifera* is evidently related to the cineole content. As previously mentioned, chemistry of *P. cineolifera* was the first of the *Prostanthera* to be published [30], which was more than a 100 years ago, but not the first to be studied. As mentioned by the author of that earlier study, Bosisto had examined *P. lasianthos* and *P. rotundifolia* but saw no commercial potential. Due to the taxonomic revisions since that time it is impossible to know of the actual identities of the specimens examined by Bosisto but our data (Table 2) include a specimen of *P. rotundifolia* (ROA-2a) that is chemically very similar to *Eucalyptus globulus* Labill. [33], which was something that Bosisto had plenty of already.

Nevertheless, the study of *P. cineolifera* was possibly motivated by its widespread use as a fly repellent. At the time, it did not have a taxonomic rank, so the authors characterised the species and the essential oil in the same study, but did not describe any fly repellent compounds. However, in the current study, chemical analysis of three specimens (CIA-1–CIA-3) identified moderate amounts of β-caryophyllene oxide (2.4–7.3%; Table 2), which is a potent mosquito repellent [34] and may also be a fly repellent. Due to the high yield of volatiles of this species, a moderate relative percentage of β-caryophyllene oxide effectively gives a high net yield, making this observation plausible. However, the combination of β-caryophyllene oxide and prostantherol in the profile serves as a useful diagnostic chemical criterion for this taxon, which is supported by the separate clade branching in Figure 2 and Figure 3 (Branch D2 of Figure 3).

The final species that is conditionally mistaken for *P. ovalifolia* is *P. prunelloides* R.Br., but it is not considered affiliated as strongly as other taxa. This is another species that displays strong variation in leaf shape. A specimen found growing on Mt Dangar of the Hunter Valley region (PRS-1, Sandy Hollow) was misidentified as *P. ovalifolia* and when we resampled it the reasoning became clear. In higher altitudes the leaves become narrow and elongate but maintain the undulation of leaf margins characteristic of the typical specimen. This contrasts with the spherical shape of leaves on specimens in the surrounding plains. Similar to the effects observed on *P. lanceolata* (LAA), morpohological variation may be related to root anchoring. Mature specimens of *P. prunelloides* in the lowlands are found adjacent to sandstone boulders that serve as ‘root runners’ for the species, conserving moisture and insulating against the hot sun to conserve the shallow root systems of the species. These same specimens were those sampled by Penfold almost a hundred years earlier.

Penfold did not publish anything specifically on *Prostanthera* but instead he distilled essential oil from *P. prunelloides*, kept it in his laboratory and decided not to study it any further, probably because of challenges in identifying maaliol, which was not a well-known compound at the time. In 1962, Hellyer mistook this essential oil as derived from *Eriostemon myoporoides* DC (now *Philotheca myoporoides* (DC) Bayly.) and claimed that, after many years of standing on a shelf in Penfold’s laboratory, the essential oil started to grow crystals that Penfold believed to be ledol, due to the similarity in shape and appearance, but they were in fact made from maaliol [33]. However, Penfold had already published his study of *E. myoporoides* (*P. myoporoides*) much earlier, in which he correctly described the essential oil composition, declaring no traces of maaliol [35].

During field work in 2012 to collect samples for the current study, the Mt Dangar specimen of *P. prunelloides* was found growing adjacent to *E. myoporoides* (*P. myoporoides*). Similarly, *P. cineolifera* (CIA-2) was also found growing with *E. myoporoides* at Wingen Maide NR. On both occasions, we sampled *E. myoporoides* to isolate the maaliol reported by Hellyer [33], but failed to detect any after both attempts. Hence, the most probable explanation is that Hellyer had incorrectly identified Penfold’s collection. This was one of two errors in that paper, which will be explained in the next section.

Nevertheless, maaliol was first identified in 1908 by an anonymous author [36] in the resinous exudate of a Samoan medicinal species *Canarium samoense* Engl., then soon after in *Valeriana officinalis* L., but the etymology of this sesquiterpene is unclear. It was for some time believed to be synonymous with ledol and this was possibility the thinking of Penfold when he observed it in the *P. prunelloides* essential oil. Maaliol and kessane rich essential oils are produced from the Indian ayurvedic plant *Valeriana jatamansi* Jones ex Roxb. [37]. One of the chemotypes of *Valeriana walichii* DC also expresses a significant amount of maaliol, and specifically the maaliol content was demonstrated to contribute to antinociceptic activity by inhibition of prostaglandin synthesis [38]. With consideration to Hellyer’s error, it is evident that within Australia maaliol is only found in significant yields in species of *Prostanthera*, although some amounts were identified in *Phebalium squamulosum* subsp. *angustifolium* Paul G. Wilson [39].

The maaliol-rich essential oil of *P. prunelloides* also includes a significant relative quantity of *Z*-dihydroagarofuran (Table 3) which reinforces its relatedness to *P. lanceolata*. However, according to the molecular phylogeny by Wilson et al. [29], *P. prunelloides* is closely related to *P. ringens* Benth. (RIS-1, -2 and -3) from section Klanderia, which is another maaliol-rich species (Table 3) that also expresses prostantherol. However, the closest chemical relative of *P. prunelloides* is *P. cuneata* (CUA-1, Table 3), which expresses a near identical chemical profile. Furthermore, like *P. ringens*, *P. lithospermoides* F.Muell. also expresses a significant amount of maaliol (LIS-1, Table 3) with no *Z*-dihydroagarofuran. The section placement of *P. lithospermoides* has not yet been published.

### 3.4. Taxa Afiliated with Prostanthera Rotundifolia

In the current study, the species included that are often incorrectly identified as *P. rotundifolia* are: the two forgotten species *P. cotinifolia* A.Cunn. ex Benth. (COA-1) and *P. latifolia* (LAIA-1a, -1b), and the new species, *P.* sp. Blue Mountains (BEM-1), *P.* sp. Ulan (ULN-1, ‘The Drip’), and *P.* sp. Barren Mountain (BNM-1, -2 and -3). In the current study, three specimens of actual *P. rotundifolia* were also sampled, one from Tasmania (ROA-1) and two from Victoria (ROA-2a and -2b).

The current study constitutes the first chemical analysis of essential oil from *P.* sp. Blue Mountains (BEM), *P.* sp. Barren Mountain (BNM, above) and *P. cotinifolia* (Table 2). Despite the strong morphological similarity of BNM and BEM the chemical character of essential oils displays minimal overlap of major components, with only 1,8-cineole in common. As previously mentioned, the prostantheral + ledol composition of BNM makes it comparable to *P. petraea* (Table 5, Figure 6), which contrasts with BEM that is comprised by nearly 50% of *Z*-dihydroagarofuran. The globulol-rich essential oil of *P. cotinifolia* was the most like the actual *P. rotundifolia*, but the comparison is still poor. This is because variation within *P. rotundifolia* was pronounced, i.e., kessane was present in two specimens only, epiglobulol in one specimen that also had nearly 30% globulol, compared to traces in the other two. Furthermore, ledol was the dominant sesquiterpene in one and prostantherol another. This pronounced chemical variation of hydroazulenic and hydronapthalenic scaffolds was realised by Southwell and Tucker (globulol vs. viridiflorol) during the course of their study of *P. lanceolata* [17]. This pronounced chemical variation is also evident in the dendrograms of Figure 2 and Figure 3. However, what is surprising is that the cluster that includes many representatives of the *P. rotundifolia* aggregate in Figure 3 (branch D2), including ROA-1, -2a, and -2b; LAIA-1 and -2; ULN-1; and the *P. cineolifera* specimens.

The dataset also conveys some degree of geographical chemical convergence of species that are clearly distinct taxa according to morphology. This has also been observed in South American taxa [40]. For example, the closest chemical relative to ULN-1, according to Figure 3 branch D2, is COA-1, which were collected <150 km apart. While COA-1 has a published and accepted name (i.e., *P. cotinifolia*), ULN-1 was recognised by Althofer but a revision was never published.

### 3.5. Chemical Groups in Prostanthera

A second PCA analysis (Figure 5) for species outside of the *P. ovalifolia* complex (including the ambiguously placed *P. prunelloides*) created four chemical groups that efficiently represent the chemical diversity of *Prostanthera*. As can be seen in the loading plot (Figure 5B) group 1 is characterised by high yields of prostantherol, group 2 by a predominantly monoterpenoid composition, including components such as α-terpinyl acetate, sabinene and α-phellandrene, group 3 by high maaliol content and group 4 by globulol, ledol and 1,8-cineole. The asterisk ‘*’ represents *P.* sp. Blue Mountains (BEM) and *P. aspalathoides* (ASS), which are two species that include Z-dihydroagarofuran and represent a fifth category, which correlates to group C in Figure 4 or Branch B in Figure 3. Thus, the fifth category is the kessane and *Z*-dihydroagarofuran yielding species that are expressed abundantly from *P. lanceolata*. Since *Z*-dihydroagarofuran was first described nearly a decade after the study of Conn [27] it was not reported in his wide geographic study of *P. aspalathoides* and so is reported here for the first time. One other species expressed *Z*-dihydroagarofuran, which is a new species that we have phrase named *P. sp*. Gibraltar Range.

Similar to groups 3 and 4 in the PCA of Figure 5, branching patterns in the Figure 6 dendrogram conveys secondary branches specifically for maaliol rich species (branch B) and *P. incisa* (branch C), respectively. In the current study, *P. incisa* (INA-1a to -1e and -2) was the next species after *P. lanceolata* with high sampling numbers, with six replicates. The strongly incised juvenile leaves of *P. lanceolata* resemble those from *P. incisa* to an extent.

Australian botanists generally regard *P. incisa* to be synonymous with *P. sieberi* but it is regarded as an accepted name on the plant list [11]. Hellyer also studied *P. sieberi* Benth. [33], which he authenticated by comparison to essential oil of *Eucalyptus globulus*. Hellyer’s globulol-rich chemical profile of *P. sieberi* [33] markedly differs from the profile of *P. incisa* of the current study (INA, Table 3 and Figure 5), which yielded a predominantly monoterpenoid essential oil comprised by 1,8-cineole, α-phellandrene, α-terpineol acetate and the sesquiterpene cadinol isomers, but no globulol. Unlike the *P. rotundifolia* group, the chemical profile of *P. incisa* remained relatively consistent. While the rank of *P. sieberi* remains controversial, the current analysis questions the proposed synonymity. However, Hellyer also studied *P. rotundifolia* from Tasmania and reported a high yield of globulol, giving a chemical profile suspiciously similar to the description he gave for *P. sieberi*. Thus, Hellyer’s joint study of Tasmanian *P. rotundifolia* with *P. sieberi* and the coincidental similarity of their essential oil profiles [33] beckons for a second analysis.

All other species are outside of these clusters. Briefly, *P. suborbicularis* C.T.White and W.D.Francis (SUS-1) and *P. striatiflora* (STA-1) are similar, expressing high relative amounts of spathulenol, globulol and α-copaene. The two phrase named specimens of *P. sp.* Baking Board (BGB-1 and -2) and *P. caerulea* R.Br. (CAA-1) are predominantly monoterpenoid with >83% of 1,8-cineole + β-pinene. The phrase named new species *P.* sp. Minyon Falls (MNF-1: J.B.Williams NE61356) expressed a high amount of caryophyllene and caryophyllene oxide, potentially making it an insect repellent like *P. cineolifera*. The phrase-named *P.* sp. Thredbo River (TOR-1) produced an essential oil characteristic of the *P. lasianthos* group [8], which includes 1,8-cineole, linalool and linalyl acetate. A new species affiliated with *P. melissifolia* F.Muell, known under the phrase name *P. sp.* Dandahra Creek, produced an essential oil very similar to that of *Eucalyptus globulus* and one of the chemical variants of *P. rotundifolia* observed in the current study. Lastly, the three phrase-named specimens of *P*. sp. Olney State Forest (OSF-1, -2 and -3, Table 4) are potentially representative of two distinct taxa, which will require reassessment of morphology for confirmation. This is evident from the pronounced difference in essential oil yield (0.1 vs. 0.5–0.9%) and chemical character, wherein OSF-3 demonstrates 0.1% yield of essential oil with a predominantly sesquiterpeneoid character. In this last example, the signs of phenoplasticity of volatiles are not hitherto evident, which conveys that this difference may not be an environmentally affected chemotype.

### 3.6. The Philosophy of Chemophenetics

During the paradigm of chemotaxonomy, when chemical profiles were considered as an absolute taxonomic criterion, phenoplasticity would have seemed like a contradiction. During that era, many scholars were proposing mechanisms to control this variability and ensure reproducibility of chemical profiles, which involved controlling for sampling time of the day to eliminate diurnal effects, season to eliminate influences of temperature and rainfall, and storage methods to eliminate artefacts of postharvest processing. However, efforts to control the factors that created variability were defeated by expansion of study into other taxa, and over time the school of chemotaxonomy was quashed by irreproducibility in some groups, including *Prostanthera*, which boasts many unpublished studies (file drawer effect).

Nevertheless, chemical studies continue to provide leads that guide or persuade morphological scrutiny of groups. Hence, in the modern paradigm chemical characterisation continues to be regarded as an informative tool but is no longer considered by any means an absolute taxonomic criterion. In this regard, the loaded and strongly controversial term ‘chemotaxonomy’ needed to be replaced to continue this line of enquiry without facing the criticism of researchers who are less informed of modern enlightenment in this field. Hence, in the modern paradigm chemical exploration across and within taxa, or heterogeneous aggregates and sections, is known as ‘chemophenetics’ [41,42].

Chemophenetic studies are often complemented by observed morphological, geographical, or molecular patterns. As a complement to other lines of evidence in defining a species [43], chemical data must be identified as representing four possible scenarios: (1) INTRA-specific variability-A. Distinct chemical clusters within a single taxon that are denoted as chemotypes; (2) INTRA-specific variability-B. Nonclustering variability within a single taxon that is denoted as flamboyant inter-specimen chemical expression differences; (3) INTER-specific variability. Distinct chemical clusters across taxa that characterises species distinctness; and (4) INTER-specific convergence. Distinct chemical convergent clusters that comprise two or more taxa, which may be a consequence of conservative chemical expression in related taxa, or convergence of distantly related or unrelated taxa.

Chemotypes can be created by the phenomenon of phenotypic plasticity [8], where expression patterns and levels of volatiles are influenced by several environmental factors, including aridity or moisture retention in soils, the frequency of ephemeralism (short vs. long wet and dry cycles) and so forth. However, usually the identity and pattern of phenoplastic volatiles is shared with other specimens of the same species within the immediate vegetative population (except in the case of random emergent chemotypes [44]). As previously mentioned, phenoplasticity was observed in populations of species allied to *P. lasianthos* [8], which created distinct chemotypes. However, flamboyant expression patterns appear to characterise *P. rotundifolia* profiles in the current study, from the Tasmanian population (ROA-2a and -2b). Hence, since phenoplasticity is active in *Prostanthera* it makes sense to elucidate this phenomenon to create a clear picture of variation due to taxa verses variation due to plasticity.

## 4. Conclusions

While chemophenetics can reveal previously unknown relationships within heterogeneous species aggregates or reiterate observations made in current or earlier times, the flamboyant expression of phenoplastic compounds in some species means that patterns are not easily perceptible to scientists from other disciplines, or the general community. Nevertheless, to a phytochemist, the patterns are clear. The data show that some species are related by the presence of sesquiterpene ethers, *Z*-dihydroagarofuran and kessane. Some are related by decahydroazulenic compounds, such as globulol, ledol or epiglobulol. Some are related by decahydronapthalenic compounds, such as prostantherol or maaliol.

The currently assigned heterogeneous species aggregates *P.* sp. aff. *ovalifolia* and *P.* sp. aff. *rotundifolia* are not segregated according to their phytochemistry. Rather, individual taxa are demonstrated to generally stand-alone according to individual essential oil chemical profiles. For example, *Z*-dihydroagarofuran is expressed in *P. lanceolata* (LAA: affiliated with *P. ovalifolia*) and in *P.* sp. Blue Mountains (BEM: affiliated with *P. rotundifolia*). However, both are segregated by multivariate analysis (Figure 3), except for the one specimen LAA-1 that expressed a low amount of p-cymene (Table 1). Nevertheless, by far the prevailing chemical phenotypes do not overlap.

Much evidence is in favor of environmental variables as the factor that influences phenoplasticity in the expression of volatiles. The major consequences of this will be felt in horticulture, because some degree of chemical variation will be evident in garden specimens as compared to wild specimens. In most cases, the variation will be within limits, but some species are expected to be dramatically different, such as *P. petraea*.

## Figures and Tables

**Table 1:** Chemical character of essential oils from *Prostanthera lanceolata* (LAA).

-	-	*Taxon*	LAA-1	LAA-2	LAA-3	LAA-4	LAA-5	LAA-6	LAA-7	LAA-8	LAA-9	LAA-10	LAA-11	LAA-12a	LAA-12b	LAA-12c	LAA-12d	LAA-13
-	-	Yield *	1.4	0.9	1.6	1.3	1.1	1.4	0.6	1.4	0.5	1.2	1.1	1.2	1.9	1.4	2.0	1.5
Compound Name	RI-a **	RI-b **																
α-Thujene	928	924	-	0.8	1.6	-	-	-	-	-	-	-	-	-	-	-	-	-
α-Pinene	934	932	-	0.8	2.0	0.6	-	-	-	-	-	-	-	-	-	-	-	-
Sabinene	973	969	1.1	0.8	1.8	0.7	0.4	-	-	1.1	-	-	-	0.6	0.8	0.7	0.8	-
β-Pinene	979	974	1.0	2.1	5.8	1.6	0.6	-	-	1.0	-	-	-	0.4	0.6	0.5	0.7	0.7
α-Phellandrene	1005	1002	-	0.4	0.4	0.3	2.1	0.8	-	-	-	-	-	1.0	0.7	0.8	1.1	1.3
p-Cymene	1025	1020	2.5	1.5	-	2.8	6.2	12.1	1.7	8.8	2.8	1.7	5.8	2.2	1.9	3.8	3.2	11.2
Limonene	1029	1024	-	-	0.8	0.5	0.7	-	-	-	-	0.7	-	0.5	-	0.5	-	3.3
1,8-Cineole	1034	1031	27.7	20.5	21.4	21.9	21.5	53.6	17.0	65.9	13.8	17.7	43.9	24.3	28.7	28.7	41.0	28.8
Linalool	1101	1100	-	0.4	-	-	-	-	-	-	-	-	-	-	-	-	-	-
E-Thujone	1115	1112	-	-	-	0.8	-	0.7	-	-	-	-	-	-	-	-	-	-
E-Pinocarveol	1136	1135	-	-	-	-	-	-	-	-	-	-	-	-	0.3	-	0.5	-
Santalone	1179	1177	-	-	-	0.3	-	-	-	-	-	-	-	-	-	-	-	-
Terpinen-4-ol	1180	1174	-	0.3	-	0.4	-	0.7	-	-	-	-	0.5	0.3	0.4	0.4	0.5	-
α-Terpineol	1192	1186	1.9	1.2	2.4	0.4	1.2	-	-	-	-	-	3.9	0.9	-	1.0	1.7	-
Myrtenal	1201	1195	-	0.7	0.6	1.2	-	-	-	-	-	-	0.5	0.4	0.5	0.4	0.4	-
Terpinyl acetate	1328	1316	-	-	-	-	-	-	-	-	-	-	-	-	0.6	-	-	-
α-terpineol acetate	1349	1344	-	-	-	1.8	0.7	-	-	-	-	-	-	-	4.0	1.1	-	-
E-Caryophyllene	1419	1417	-	-	-	-	0.8	0.6	-	-	-	-	2.0	-	-	-	-	-
Dehydroaromadendrane	1466	1460	-	-	-	-	-	-	-	-	-	-	-	0.5	1.1	0.5	-	-
α-Amorphene	1481	1485	-	-	-	-	-	-	-	-	-	-	-	1.0	1.1	1.0	-	-
Z-β-Guaiene	1491	1492	-	-	0.6	-	-	-	-	-	-	-	1.9	-	-	-	-	1.6
Bicyclogermacrene	1504	1500	0.9	-	-	-	10.8	4.5	-	0.9	-	-	10.7	0.3	0.8	-	-	-
Z-Dihydroagarofuran	1525	1519	59.4	14.9	-	18.9	-	19.0	-	16.5	59.2	42.1	21.5	2.8	9.6	-	23.4	28.4
δ-Cadinene	1527	1522	-	0.7	-	0.3	-	-	-	-	-	-	-	0.5	-	-	-	-
Kessane	1532	1529	-	24.4	43.7	28.8	37.8	-	76.9	-	17.3	31.8	-	32.2	13.9	34.7	10.0	5.1
α-Cadinene	1535	1537	-	0.4	-	0.5	0.7	1.6	-	-	-	-	-	1.6	0.7	1.6	0.5	-
Epiglobulol	1573	NMR	-	-	-	-	2.0	-	-	2.9	-	-	0.4	-	6.8	-	-	-
Spathulenol	1578	1577	-	-	-	-	-	1.9	-	-	-	-	2.5	-	-	-	-	-
Caryophyllene oxide	1584	1582	-	-	-	-	-	-	-	-	-	-	1.4	-	-	-	-	-
Globulol	1590	1590	-	19.5	13.3	14.7	-	0.8	-	-	-	3.1	1.9	27.9	9.1	23.4	6.9	4.8
Viridiflorol	1601	1592	-	-	-	0.7	-	-	-	-	-	-	-	-	-	-	-	-
Prostantherol	1602	NMR	1.3	1.1	-	-	3.1	1.2	2.9	-	1.3	0.5	1.9	1.4	16.0	-	8.0	14.7
Ledol	1610	1602	-	-	-	-	3.3	2.6	-	-	5.5	2.2	-	-	1.2	-	0.7	-
Cubenol	1619	1618	-	9.1	5.4	2.7	-	-	-	-	-	-	-	-	-	-	-	-
Alloaromadendrene epoxide	1638	1632	0.7	0.3	-	-	1.4	-	-	2.8	-	-	1.1	-	-	-	-	-
Cadinol-epi-alpha	1648	1638	-	-	-	-	-	-	-	-	-	-	-	1.1	0.4	0.5	0.4	-
α-Cadinol	1661	1652	-	-	-	-	-	-	-	-	-	-	-	0.4	-	0.4	-	-
n.d.	1748	n/a	3.4	-	-	-	6.7	-	1.5	-	-	-	-	-	0.6	-	-	-

* Yield is in % g/g fresh leaf weight. ** RI-a is the calculated retention index and RI-b is the published reference value of the retention index.

**Table 2:** Chemical character of essential oils from taxa affiliated with *P. ovalifolia*, and *P. rotundifolia*. OVA = *P. ovalifolia* (actual), ROA = *P. rotundifolia* (actual), CIA = *P. cineolifera*, LAIA = *P. latifolia*, BNM = *P.* sp. Barren Mountain, BEM = *P.* sp. Blue Mountains, COA = *P. cotinifolia*, ULN = *P. sp.* Ulan, DAC = *P*. sp. Dandarha Creek.

-	-	*Taxon*	OVA-1	CIA-1	CIA-2	CIA-3	LAIA-1a	LAIA-1b	BNM-1	BNM-2	BNM-3	BEM-1	COA-1	ROA-1	ROA-2a	ROA-2b	ULN-1	DAC-1
-	-	Yield *	0.6	0.2	0.8	1.1	0.9	0.7	2.4	2.7	2.1	1.5	1.3	1.1	1.5	0.6	0.9	1.3
Compound Name	RI-a **	RI-b **																
α-Thujene	928	924	0.3	-	5.2	-	-	-	-	-	-	-	2.8	-	-	-	-	-
α-Pinene	934	932	11.6	-	0.8	0.7	5.5	13.9	-	-	-	-	1.5	0.7	0.4	0.5	-	0.4
Camphene	951	946	2.0	-	-	-	0.8	2.2	-	-	-	-	-	-	-	-	-	-
Verbenene	956	961	0.5	-	-	-	0.9	0.5	-	-	-	-	-	-	-	-	-	-
Sabinene	973	969	-	-	0.7	-	0.3	1.0	-	-	-	-	0.6	1.5	0.4	1.0	-	0.5
β-Pinene	979	974	1.4	0.5	1.1	1.5	0.3	1.5	-	-	-	-	1.5	0.9	0.5	0.8	0.8	0.6
Myrcene	987	988	0.4	-	-	-	0.3	0.4	-	-	-	-	-	-	-	-	-	-
α-Phellandrene	1005	1002	1.5	-	1.1	-	2.1	1.3	1.5	1.1	1.3	-	-	4.2	0.3	3.6	-	2.0
p-Cymene	1025	1020	6.2	12.7	17.5	19.3	7.6	4.2	3.4	2.6	3.0	1.2	7.2	4.5	3.1	4.7	2.0	9.5
Limonene	1029	1024	0.4	-	-	1.1	0.4	0.6	-	-	-	-	1.3	-	-	1.8	-	1.5
1,8-Cineole	1034	1031	22.6	38.8	49.7	64.2	11.9	30.3	11.5	8.7	10.1	30.2	63.6	39.6	29.9	33.0	65.6	31.8
p-Cymenene	1091	1089	-	-	-	-	0.4	0.7	-	-	-	-	-	-	-	-	-	-
Linalool	1101	1100	0.2	-	-	-	-	-	0.8	0.6	0.7	-	-	-	-	-	-	-
E-Thujone	1115	1112	1.2	1.2	1.6	-	-	-	-	-	-	-	-	-	-	-	-	-
E-Pinocarveol	1136	1135	1.0	-	-	-	-	1.0	-	-	-	-	0.9	-	-	-	-	-
Z-Verbenol	1143	1137	-	-	-	-	3.5	-	-	-	-	-	-	-	0.4	-	-	-
E-Verbenol	1147	1140	-	-	-	-	15.4	5.2	-	-	-	-	-	-	-	-	-	-
Camphor	1148	1150	5.5	-	-	-	-	-	-	-	-	-	-	-	-	-	-	-
Nonadienal	1152	1150	0.9	-	-	-	-	-	-	-	-	-	-	-	-	-	-	-
Borneol	1169	1170	8.4	-	-	-	4.1	5.2	-	-	-	-	0.9	-	-	-	-	-
Santalone	1179	1177	-	-	-	-	-	0.4	-	-	-	-	1.2	-	-	-	-	-
Terpinen-4-ol	1180	1174	0.4	0.4	0.9	-	-	-	-	-	-	-	-	-	0.3	0.4	-	-
α-Terpineol	1192	1186	3.7	0.4	1.3	-	1.0	2.7	-	-	-	-	1.8	0.4	0.9	0.7	-	-
Myrtenal	1201	1195	0.5	0.3	-	-	0.3	0.8	-	-	-	-	0.9	-	-	-	-	-
Verbenone	1213	1204	3.8	-	-	-	0.5	3.2	-	-	-	-	-	-	-	-	-	-
Linalyl acetate	1256	1254	0.2	-	-	-	-	-	1.5	1.2	1.4	-	-	-	-	-	-	-
Bornyl acetate	1287	1288	12.9	-	-	-	6.2	4.4	-	-	-	-	-	-	-	-	-	-
Thymol	1295	1289	-	0.5	-	-	0.5	0.4	-	-	-	-	-	-	0.3	-	-	-
Terpinyl acetate	1328	1316	-	-	0.9	-	-	0.6	-	-	-	-	-	-	0.3	0.6	-	-
α-terpineol acetate	1349	1344	-	-	-	-	0.5	-	-	-	-	0.6	-	3.1	1.4	4.6	-	-
α-Copaene	1373	1374	0.5	-	-	-	-	-	-	-	-	-	-	-	-	-	-	-
E-Caryophyllene	1419	1417	0.4	-	-	-	-	-	-	-	-	-	-	2.3	-	-	-	-
Aromandendrene	1443	1439	-	-	-	-	0.3	-	-	-	-	-	-	0.4	-	-	0.8	-
Alloaromadendrene	1462	1458	-	-	-	-	-	-	-	-	-	0.7	-	-	2.0	-	1.3	-
Dehydroaromadendrane	1466	1460	-	0.3	-	-	-	1.3	2.1	1.6	1.9	0.9	-	4.6	-	1.3	-	-
α-Amorphene	1481	1485	-	-	-	-	-	-	-	-	-	2.1	-	2.6	-	2.6	-	-
Z-β-Guaiene	1491	1492	-	-	-	-	-	-	-	-	-	1.7	-	-	0.4	7.2	1.8	-
Bicyclogermacrene	1504	1500	3.5	2.3	1.4	0.8	-	2.6	2.0	1.5	1.8	-	0.8	1.9	0.4	1.7	-	1.3
Z-Dihydroagarofuran	1525	1519	-	-	-	-	-	-	-	-	-	48.8	-	-	-	-	-	1.2
δ-Cadinene	1527	1522	-	-	-	-	-	1.0	-	-	-	-	-	-	-	-	-	-
Kessane	1532	1529	-	-	-	-	16.7	5.7	2.5	1.9	2.2	-	-	11.0	3.6	-	-	8.3
α-Cadinene	1535	1537	-	-	-	-	0.3	-	-	-	-	-	-	-	-	-	-	-
Maaliol	1566	NMR	-	-	-	-	-	-	-	-	-	0.9	-	-	0.7	-	-	-
Epiglobulol	1573	NMR	0.3	-	-	-	-	-	-	-	-	-	-	-	18.4	-	5.9	-
Spathulenol	1578	1577	-	-	-	-	-	0.3	-	-	-	0.5	2.7	-	0.4	-	-	-
Caryophyllene oxide	1584	1582	1.1	7.3	3.8	2.4	-	-	-	-	-	0.8	-	-	-	-	1.3	1.0
Globulol	1590	1590	0.7	1.2	-	-	20.2	5.6	-	-	-	7.2	12.1	2.3	29.5	0.8	1.8	41.2
Viridiflorol	1601	1592	-	-	-	-	-	-	-	-	-	1.7	-	-	2.3	0.7	1.5	-
Prostantherol	1602	NMR	2.9	27.0	13.8	9.9	-	0.7	49.0	61.2	55.1	1.1	-	3.2	0.4	28.9	6.8	-
Ledol	1610	1602	5.0	1.1	-	-	-	1.4	25.7	19.6	22.7	-	-	16.7	3.7	2.8	-	-
Cubenol	1619	1618	-	-	-	-	-	-	-	-	-	-	-	-	-	0.9	-	-
Alloaromadendrene epoxide	1638	1632	-	5.9	-	-	-	-	-	-	-	-	-	-	-	-	2.0	-
Caryophylla-diene-ol	1642	1644	-	-	-	-	-	-	-	-	-	-	-	-	-	-	-	0.6
Cadinol-epi-alpha	1648	1638	-	-	-	-	-	-	-	-	-	1.0	-	-	-	-	-	-
α-Cadinol	1661	1652	-	-	-	-	-	-	-	-	-	0.5	-	-	-	-	-	-
n.d.	1748	n/a	-	-	-	-	-	0.5	-	-	-	-	-	-	-	-	1.8	-
Squamulosone	1774	NMR	-	-	-	-	-	-	-	-	-	-	-	-	-	1.4	-	-
n.d.	1818	n/a	-	-	-	-	-	-	-	-	-	-	-	-	-	-	5.2	-
n.d.	1825	n/a	-	-	-	-	-	-	-	-	-	-	-	-	-	-	1.2	-

* Yield is in % g/g fresh leaf weight. ** RI-a is the calculated retention index and RI-b is the published reference value of the retention index.

**Table 3:** Chemical character of essential oils from miscellaneous species of *Prostanthera*. LIS = *P. lithospermoides*, CUA = *P. cuneata*, PRS = *P. prunelloides*, RIS = *P. ringens,* INA = *P. incisa*.

-	-	*Taxon*	LIS-1	CUA-1	PRS-1	PRS-3	PRS-2	RIS-1	RIS-2	RIS-3	INA-1a	INA-1b	INA-1c	INA-1d	INA-1e	INA-2
-	-	Yield *	1.4	3.2	2.1	2.2	2.5	4.1	3.8	4.2	0.5	1.0	1.5	0.9	1.2	0.8
Compound Name	RI-a **	RI-b **														
α-Pinene	934	932	1.0	3.0	-	0.4	0.5	1.8	1.2	2.0	-	1.0	2.7	0.7	1.9	1.8
Sabinene	973	969	-	0.8	-	-	-	-	-	-	1.3	2.8	3.8	2.3	3.7	6.1
β-Pinene	979	974	1.5	6.9	-	-	0.1	2.8	1.6	1.5	1.8	2.9	6.4	3.1	4.9	5.9
Myrcene	987	988	-	-	-	-	-	-	-	-	0.7	1.1	1.9	0.9	1.0	-
α-Phellandrene	1005	1002	-	-	-	-	-	-	-	-	5.1	12.0	12.9	6.4	10.2	1.8
p-Cymene	1025	1020	1.0	0.6	-	0.4	0.2	3.4	3.5	2.8	2.6	5.1	4.3	2.5	2.9	1.6
Limonene	1029	1024	-	1.5	-	-	-	0.7	1.0	1.5	2.9	-	1.8	-	-	3.5
1,8-Cineole	1034	1031	-	24.2	24.2	34.6	33.0	21.1	18.9	16.2	36.1	44.4	43.5	41.3	49.7	50.9
E-Thujone	1115	1112	-	0.6	-	-	-	-	-	-	-	-	-	-	-	-
Camphor	1148	1150	-	-	-	-	-	0.4	0.3	0.5	-	-	-	-	-	-
Nonadienal	1152	1150	-	-	-	-	-	2.1	3.0	1.5	-	-	-	-	-	-
Pinocarvone	1160	1160	-	0.7	-	-	-	0.7	0.1	0.9	-	-	-	-	-	-
Terpinen-4-ol	1180	1174	-	0.7	-	-	-	-	-	-	1.5	0.8	0.9	1.1	0.7	-
α-Terpineol	1192	1186	-	-	-	0.5	0.1	-	-	-	1.6	0.5	0.4	0.7	0.9	-
Myrtenal	1201	1195	1.9	1.6	-	-	-	1.9	2.5	3.9	-	-	-	-	-	-
Verbenone	1213	1204	-	1.9	-	-	-	0.4	0.1	0.8	-	-	-	-	-	-
Hexanoic acid, 1-methyl butyl ester	1216	n.f	-	-	-	-	-	0.6	0.8	0.7	-	-	-	-	-	-
Terpinyl acetate	1328	1316	-	-	-	-	-	-	-	-	1.3	1.4	0.8	2.0	0.8	1.3
α-Terpineol acetate	1349	1344	-	-	-	-	-	-	-	-	19.1	9.9	9.4	16.0	8.7	12.5
Aromandendrene	1443	1439	-	-	-	1.1	-	-	-	-	-	-	-	-	-	-
Alloaromadendrene	1462	1458	-	-	-	0.9	1.5	1.3	3.8	2.8	-	-	-	-	-	-
Germacrene D	1486	1484	-	1.6	-	-	-	-	-	-	-	-	-	-	-	-
β-Selinene	1489	1489	-	0.8	-	-	-	-	-	-	-	-	-	-	-	-
γ-Amorphene	1495	1495	-	0.8	-	-	-	-	-	-	-	-	-	-	-	-
Bicyclogermacrene	1504	1500	-	0.8	1.5	1.6	3.1	-	-	-	-	-	-	-	-	-
Z-Dihydroagarofuran	1525	1519	-	15.8	39.0	23.4	23.5	-	-	-	-	-	-	-	-	-
δ-Cadinene	1527	1522	-	-	-	-	-	-	-	-	3.4	2.5	1.6	3.1	2.0	-
Kessane	1532	1529	2.9	-	-	-	-	-	-	-	-	-	-	-	-	-
α-Cadinene	1535	1537	4.8	-	-	-	-	-	-	-	2.5	1.5	1.0	2.5	1.1	-
10-Epicubebol	1542	1533	-	-	-	-	-	-	-	-	0.6	0.7	-	0.5	0.5	-
Maaliol	1566	NMR	70.0	34.5	33.5	32.3	36.3	38.2	40.7	40.9	-	-	-	-	-	-
Epiglobulol	1573	NMR	-	-	-	-	-	0.5	0.3	0.5	1.2	1.4	0.6	1.3	1.3	1.1
Spathulenol	1578	1577	2.0	1.0	-	-	-	-	-	-	-	-	-	-	-	-
Globulol	1590	1590	12.8	1.0	2.0	2.8	1.6	1.1	1.1	1.1	-	-	-	-	-	-
Viridiflorol	1601	1592	2.1	-	-	-	-	-	-	-	-	-	-	-	-	-
Prostantherol	1602	NMR	-	1.2	-	1.9	-	21.2	19.7	16.5	-	-	-	-	-	-
Ledol	1610	1602	-	-	-	-	-	1.3	0.8	4.9	1.0	0.7	0.5	0.9	0.5	0.3
Cubenol, 1,10-di-epi	1622	1618	-	-	-	-	-	-	-	-	1.0	0.6	0.5	0.9	0.5	0.6
Cadinol-epi-alpha	1648	1638	-	-	-	-	-	-	-	-	12.0	8.2	5.4	10.8	6.8	9.4
α-Cadinol	1661	1652	-	-	-	-	-	-	-	-	4.3	2.5	1.7	3.1	1.8	3.2

* Yield is in % g/g fresh leaf weight. ** RI-a is the calculated retention index and RI-b is the published reference value of the retention index.

**Table 4:** Chemical character of essential oils from maaliol producing species, and *P. incisa* from Blue Mountains. Yield is in % g/g fresh leaf weight, AI is arithmetic index and Pub. AI is the published reference value of arithmetic index. SUS = *P. suborbicularis*, STA = *P. striatiflora*, MNF = *P.* sp. Minyon Falls, OSF = *P.* sp. Olney State Forest, CAA = *P. caerulea*, BGB = *P.* sp. Baking Board, TOR = *P.* sp. Thredbo River, GRR, *P.* sp. Gibraltar Range, ASS = *P. aspalathoides*, PIA = *P*. sp. Piliga.

-	-	*Taxon*	SUS-1	STA-1	MNF-1	BGB-1	BGB-2	GRR-1	PIA-1	ASS-1	TOR-1	OSF-1	OSF-2	OSF-3	CAA-1
-	-	Yield *	0.1	0.1	0.1	0.1	0.1	0.1	0.1	1.5	0.1	0.9	0.5	0.1	0.8
Compound Name	RI-a **	RI-b **													
2-Hexanol	-	-	-	-	-	-	-	-	-	-	-	-	-	2.1	-
2-Hexenal, (E)-	-	-	-	-	-	-	-	-	-	-	-	-	-	7.9	-
1-Hexanol	-	-	-	-	-	-	-	-	-	-	-	-	-	2.1	-
α-Thujene	928	924	-	-	-	-	-	-	-	-	-	1.0	0.6	-	-
α-Pinene	934	932	-	-	-	1.7	1.4	2.0	5.6	6.8	1.9	3.2	1.1	-	6.3
Camphene	951	946	-	-	-	-	-	-	-	-	-	1.2	-	-	-
Verbenene	956	961	-	-	-	-	-	-	-	-	-	2.9	1.2	-	-
Sabinene	973	969	-	-	-	-	-	-	-	0.9	0.4	-	-	-	-
β-Pinene	979	974	0.5	-	-	3.2	1.1	-	0.9	5.9	2.8	0.4	0.7	-	12.0
Myrcene	987	988	-	-	-	-	-	-	-	-	1.1	-	-	-	-
α-Phellandrene	1005	1002	-	-	-	-	1.0	-	0.8	-	1.2	1.4	0.5	-	-
p-Cymene	1025	1020	-	-	-	1.1	1.6	-	0.8	1.7	3.3	10.7	2.3	9.6	1.7
Limonene	1029	1024	-	-	-	4.4	3.2	1.2	2.3	1.6	2.8	0.5	-	-	3.5
1,8-Cineole	1034	1031	-	-	-	82.8	82.1	66.9	42.5	42.0	38.5	30.6	51.8	5.5	72.9
p-Cymenene	1091	1089	-	-	-	-	-	-	1.3	-	-	0.9	0.6	-	-
Linalool	1101	1100	-	-	0.7	-	-	-	0.5	-	23.0	0.5	0.7	-	-
α-Campholenal	1129	1122	-	-	-	-	-	-	-	-	-	0.6	0.6	-	-
Z-Verbenol	1143	1137	-	-	-	-	-	-	-	-	-	2.8	3.0	3.4	-
E-Verbenol	1147	1140	-	-	-	-	-	-	-	-	-	7.8	10.7	-	-
Menth-3-en-8-ol	1152	1145	-	-	-	-	-	-	-	-	-	3.6	3.2	-	-
Camphor	1148	1150	-	-	-	-	1.7	-	-	-	-	-	-	-	-
Pinocarvone	1160	1160	-	-	0.6	-	-	-	-	-	-	0.3	-	2.0	-
Mentha-1,5-dien-8-ol—para	1170	1166	-	-	-	-	-	-	-	-	-	10.6	9.3	-	-
Santalone	1179	1177	-	-	-	-	-	-	-	-	-	-	-	-	1.3
Terpinen-4-ol	1180	1174	-	0.8	-	-	1.0	-	0.6	0.4	0.9	0.6	1.2	3.9	-
p-Cymen-8-ol	1187	1179	-	-	-	-	-	-	-	-	-	1.2	1.3	-	-
α-Terpineol	1192	1186	-	3.0	-	-	1.0	-	0.8	-	3.6	0.3	-	-	-
Myrtenal	1201	1195	-	-	-	-	-	-	-	-	-	0.4	0.6	-	-
Verbenone	1213	1204	-	-	-	-	-	-	-	-	-	1.2	1.4	-	-
Linalyl acetate	1256	1254	-	-	-	-	-	-	-	-	14.6	-	1.6	-	-
Bornyl acetate	1287	1288	0.3	-	5.7	-	-	-	-	0.5	-	5.2	4.0	-	-
Thymol	1295	1289	0.6	-	0.3	-	-	-	-	-	-	0.3	1.0	2.8	-
Terpinyl acetate	1328	1316	8.2	0.6	-	-	-	-	-	-	-	-	-	-	-
α-terpineol acetate	1349	1344	0.9	-	-	-	-	-	-	-	-	0.8	2.4	-	-
Neryl acetate	1364	1359	0.3	-	-	-	-	-	-	-	0.9	-	-	-	-
Cyclosativene	1369	1369	0.9	3.6	-	-	-	-	-	-	-	-	-	-	-
α-Copaene	1373	1374	11.3	5.5	0.4	-	-	-	-	-	-	-	-	-	-
Geranyl acetate	1380	1379	0.6	-	0.2	-	-	-	-	-	1.7	-	-	-	-
E-Caryophyllene	1419	1417	0.4	-	24.8	-	-	-	2.5	-	1.1	-	-	-	-
β-Copaene	1434	1430	0.6	-	0.4	-	-	-	-	-	-	-	-	-	-
α-Guaiene	1444	1437	-	-	0.3	-	-	-	-	-	-	-	-	-	-
Aromandendrene	1443	1439	-	-	0.6	-	-	-	-	-	-	-	-	-	-
Alloaromadendrene	1462	1458	-	-	1.3	-	-	-	0.6	-	-	-	-	-	0.7
Dehydroaromadendrane	1466	1460	-	-	0.9	-	-	-	-	-	-	-	-	-	-
α-Amorphene	1481	1485	-	-	1.7	-	-	-	0.5	-	-	-	-	-	-
Germacrene D	1486	1484	-	-	2.6	-	-	-	1.8	-	0.8	-	-	-	-
β-Selinene	1489	1489	-	-	-	-	-	3.9	-	-	-	-	-	-	-
γ-Amorphene	1495	1495	-	-	0.6	-	-	-	1.8	-	-	-	-	-	-
Bicyclogermacrene	1504	1500	39.7	-	3.9	2.8	4.3	1.7	9.6	-	0.5	-	-	-	-
α-Farnesene	1509	1505	-	-	-	-	-	-	-	-	-	-	-	2.9	-
γ-Cadinene	1519	1513	-	-	-	-	-	-	-	-	-	-	-	2.3	-
Z-Dihydroagarofuran	1525	1519	-	-	-	-	-	21.4	-	15.7	-	-	-	3.7	-
δ-Cadinene	1527	1522	-	-	1.8	-	-	-	-	-	-	-	-	3.5	-
Kessane	1532	1529	-	2.3	0.6	-	-	-	-	0.6	-	0.4	-	2.5	-
α-Cadinene	1535	1537	-	2.2	1.9	-	-	-	-	-	-	-	-	-	-
10-Epicubebol	1542	1533	0.2	-	13.1	-	-	-	-	-	-	-	-	4.0	-
Maaliol	1566	NMR	0.4	1.3	1.6	-	-	-	-	0.6	-	-	-	3.8	-
Epiglobulol	1573	NMR	2.0	0.6	-	-	-	-	3.9	-	-	-	-	-	-
Spathulenol	1578	1577	16.4	59.3	6.0	1.0	1.6	-	-	1.2	-	-	-	4.2	-
Caryophyllene oxide	1584	1582	-	-	12.1	3.0	-	-	-	-	0.4	-	-	-	-
Globulol	1590	1590	5.6	2.3	-	-	-	2.9	11.4	19.8	0.5	10.5	-	9.8	-
Viridiflorol	1601	1592	-	-	-	-	-	-	-	1.4	-	-	-	-	-
Prostantherol	1602	NMR	5.6	-	-	-	-	-	5.5	0.6	-	-	-	3.0	1.4
Ledol	1610	1602	2.2	-	5.2	-	-	-	4.1	-	-	-	-	7.8	-
Cubenol	1619	1618	2.6	2.8	-	-	-	-	0.5	-	-	-	-	1.8	-
Alloaromadendrene epoxide	1638	1632	0.8	4.9	-	-	-	-	2.0	-	-	-	-	-	-
Cubenol, 1,10-di-epi	1622	1618	-	2.1	1.6	-	-	-	-	-	-	-	-	-	-
Cadinol-epi-alpha	1648	1638	-	2.5	3.1	-	-	-	-	-	-	-	-	2.4	-
α-Cadinol	1661	1652	-	6.2	8.3	-	-	-	-	-	-	-	-	-	-
Cadilene	1680	1675	-	-	-	-	-	-	-	-	-	-	-	9.0	-

* Yield is in % g/g fresh leaf weight. ** RI-a is the calculated retention index and RI-b is the published reference value of the retention index.

**Table 5:** Essential oils from *P. petraea* (PEA).

			PEA-1a	PEA-1b	PEA-1c	PEA-1d	PEA-1e
	-	Yield *	1.7	1.9	1.2	1.1	1.6
	RI-a **	RI-b **					
α-Phellandrene	1005	1002	-	-	1.7	1.9	-
p-Cymene	1025	1020	-	0.7	2	4	-
Alloaromadendrene	1464	1458	-	0.8	0.7	-	-
Dehydroaromadendrane	1466	1460	-	0.6	-	-	-
α-Amorphene	1481	1485	0.8	0.7	2.1	1.7	1.1
γ-Amorphene	1495	1495	1.2	0.6	0.9	1.1	-
Bicyclogermacrene	1501	1500	1.6	0.9	1.2	1.2	-
Kessane	1534	1529	18.9	22.5	18.4	17.7	18.1
spathulenol	1574	1577	-	0.6	-	0.7	-
Prostantherol	1600	1600	75.6	51.7	64.6	68.5	76.9
Ledol	1604	1602	1.9	17.4	1.7	1.8	1.9
n.d.	1618	-	-	-	4.2	1.4	1.1
Caryophylla-dien-ol	1642	1644	-	1.2	-	-	0.8
n.d.	1664	-	-	-	1.3	-	-

* Yield is in % g/g fresh leaf weight. ** RI-a is the calculated retention index and RI-b is the published reference value of the retention index.

## Supplementary Materials

The following are available online at https://www.mdpi.com/2223-7747/9/11/1570/s1. Table S1: Species studied and their corresponding codes used for Figure 2, Figure 3 and Figure 4, collector’s number and location of collection. Table S2. Species, reference and code corresponding to essential oil chemical profiles taken from published data. To see the chemical profiles of these species see cited literature [supplementary: 1,2]. Figure S1: ^13^C NMR of kessane isolated from the variagated ‘Prostanthera ovalifolia’ from a cultivated specimen. Processing software is Spinworks™. Figure S2: ^13^C NMR of pure prostantherol isolated from Prostanthera petraea. Processing software is Spinworks™. Figure S3: ^13^C NMR of near pure maaliol in whole essential oil from Prostanthera lithospermoides. Processing software is Spinworks™. Figure S4: ^13^C NMR of globulol in enriched fraction of the essential oil from Prostanthera aspalathoides (collection from condenser by hexane gave globulol and Z-dihydroagarofuran). Processing software is Spinworks™. Figure S5: ^13^C NMR of epiglobulol as a major component of the whole essential oil of Tasmanian Prostanthera rotundifolia. The selected shifts (ppm) are from the epiglobulol. Processing software is Spinworks™. Figure S6: ^13^C NMR of ledol in whole essential oil from Tasmanian Prostanthera rotundifolia. The selected shifts (ppm) are for the ledol. Processing software is Spinworks™. Figure S7: ^13^C of Z-dihydroagarofuran in enriched fraction of essential oil from Prostanthera aspalathoides (collection from the condenser by hexane gave this enriched fraction). Processing software is Spinworks™. Figure S8: ^13^C NMR spectra of globulol isolated from Prostanthera sp. Piliga. Processing software is Spinworks™.
