# Supplementary material for: Prostanthera (Lamiaceae) as a ‘Cradle of Incense’: Chemophenetics of Rare Essential Oils from Both New and Forgotten Australian ‘Mint Bush’ Species
Source: Plants (Basel). 2020 Nov 13;9(11):1570. doi: 10.3390/plants9111570 (PMC7696040; doi:10.3390/plants9111570)
Supplement: Supplementary file 1 [file plants-09-01570-s001.pdf]

Supplementary Files for '*Prostanthera* (Lamiaceae) as a 'cradle of incense': Chemophenetics of rare essential oils from both new and forgotten Australian 'mint bush' species'

Table S1. Species studied and their corresponding codes used for Figures 2, 3 and 4, collector's number and location of collection.

Code	Species	Collector's Number	Location
**ASS**	*P. aspalathoides* A.Cunn. ex Benth.	N.J. Sadgrove 544	Talleeban NR, Griffith, NSW
**CAA**	*P. caerulea* R.Br.	N.J. Sadgrove 272	Winburdale NR, 20 km ENE of Bathurst, NSW
**CIA-1**	*P. cineolifera* R.T.Baker & H.G.Sm.	N.J. Sadgrove 291	'Wallaby Rocks', near Wybong, NSW
**CIA-2**	*P. cineolifera* R.T.Baker & H.G.Sm.	N.J. Sadgrove 313	Wingen Maid, N of Scone, NSW
**CIA-3**	*P. cineolifera* R.T.Baker & H.G.Sm.	N.J. Sadgrove 317	Pokolbin SF, NSW
**COA-1**	*P. cotinifolia* A.Cunn. ex Benth.	N.J. Sadgrove 543	Warrumbungle NP, NSW
**INA-1, a-e***	*P. incisa* R.Br.	J.J. Bruhl 3532	Forbes River, Werrikimbe NP, NSW
**INA-2**	*P. incisa* R.Br.	N.J. Sadgrove 274	Blue Mtns NP, 4 km SW of Katoomba, NSW
**LAA-1**	*P. lancealata* Domin	R.L. Palsson 104	Tamborine Mtn, Qld
**LAA-2**	*P. lancealata* Domin	R.L. Palsson 108	Border Ranges NP, NSW
**LAA-3**	*P. lancealata* Domin	R.L. Palsson 110	Border Ranges NP, NSW
**LAA-4**	*P. lancealata* Domin	R.L. Palsson 111	Border Ranges NP, NSW
**LAA-5**	*P. lancealata* Domin	R.L. Palsson 118	Tabbimoble Creek, NSW
**LAA-6**	*P. lancealata* Domin	R.L. Palsson 122	Middle Creek, Sherwood NR, NSW
**LAA-7**	*P. lanceolata* Domin	J.J. Bruhl 3545	Minyon Falls, Nightcap NP, NSW
**LAA-8**	*P. lanceolata* Domin	N.J. Sadgrove 239	Nymboida River, Namoi-Binderay NP, NSW
**LAA-9**	*P. lanceolata* Domin	N.J. Sadgrove 321	Hells Hole, Mt Jerusalem NP, NSW
**LAA-10**	*P. lanceolata* Domin	N.J. Sadgrove 325	Minyon Falls, Nightcap NP, NSW
**LAA-11**	*P. lanceolata* Domin	N.J. Sadgrove 455	Sherwood NR, NSW
**LAA-12, a-d***	*P. lanceolata* Domin	J.J. Bruhl 3563	Oxley Road, New England NP, NSW
**LAA-13**	*P.* sp. Ellenborough Falls	N.J. Sadgrove 423	Ellenborough Falls, NSW
**LAIA-1, -2***	*P. latifolia* (Benth.) Domin	N.J. Sadgrove 424	Rowleys Rock, Tapin Tops NP, NSW
**LIS-1**	*P. lithospermoides* F.Muell.	N.J. Sadgrove 473	Miles – Roma, Warrego Hwy, Qld
**OVA-1**	*P. ovalifolia* R.Br.	R.L. Palsson 200	Mt Stanley, Mt Castle Tower NP, Qld
**PRS-1**	*P. prunelloides* R.Br.	N.J. Sadgrove 348	Mt Dangar, W of Sandy Hollow, NSW
**PRS-2**	*P. prunelloides* R.Br.	N.J. Sadgrove 512	Near Sandy Hollow, NSW
**PRS-3**	*P. prunelloides* R.Br.	N.J. Sadgrove 516	Near Putty, NSW
**RIS-1**	*P. ringens* Benth.	N.J. Sadgrove 493	Moonie Hwy, Qld
**RIS-2**	*P. ringens* Benth.	N.J. Sadgrove 513	Moonie Hwy, Qld
**RIS-3**	*P. ringens* Benth.	N.J. Sadgrove 531	Moonie Hwy, Qld
**ROA-1**	*P. rotundifolia* R.Br.	J.R. Nevin 155	Bradys Lookout, N of Launceston, Tas.

**ROA-2, a,b***	*P. rotundifolia* R.Br.	J.R. Nevin 158	Genoa Falls, Croajingalong NP, Vic.
**SUS-1**	*P. subobicularis* C.T.White & W.D.Francis	N.J. Sadgrove 549	10 km W of Charleville, Qld
**BGB-1**	*P* sp. Baking Board	N.J. Sadgrove 475	W of Miles, Qld
**BGB-2**	*P* sp. Baking Board	N.J. Sadgrove 508	N of Miles, Qld
**BNM-1**	*P.* sp. Barren Mtn	N.J. Sadgrove 366	Barren Mtn, New England NP, NSW
**BNM-2**	*P.* sp. Barren Mtn	N.J. Sadgrove 427	Barren Mtn, New England NP, NSW
**BNM-3**	*P.* sp. Barren Mtn	N.J. Sadgrove 428	Barren Mtn, New England NP, NSW
**BEM-1**	*P.* sp. Blue Mtns	N.J. Sadgrove 542	Glow-worm Tunnel, Wollemi NP, NSW
**DAC-1**	*P.* sp. Dandahra Creek	N.J. Sadgrove 296	Mulligans Hut, Gibraltar Range NP, NSW
**GRR-1**	*P.* sp. Gibraltar Range	N.J. Sadgrove 299	Granites Lookout, Washpool NP, NSW
**OSF -1**	*P.* sp Olney S.F.	R.L. Palsson 162	Olney SF, NSW
**OSF -2**	*P.* sp Olney S.F.	R.L. Palsson 165	Olney SF, NSW
**OSF-3**	*P.* sp Olney S.F.	R.L. Palsson 172	Olney SF, NSW
**PIA-1**	*P.* sp. Pilliga	N.J. Sadgrove 378	Goonoo Goonoo SF, NSW
**TOR-1**	*P.* sp. Thredbo River	J.J. Bruhl 3371	Alpine Way, Kosciuszko NP, NSW
**ULN-1**	*P.* sp. Ulan	N.J. Sadgrove 292	The Drip, Goulburn River NP, NSW
**MNF-1**	*P. scutellarioides*	N.J. Sadgrove 324	Minyon Falls, Nightcap NP, NSW
**STA-1**	*P. striatiflora*	N.J. Sadgrove 263	Broken Hill, NSW
**CUA-1**	*P. cuneata*	1985-8646 WAK	Royal Botanic Gardens, Kew, London
**PEA-1, a-e***	*P. petraea*	N.J. Sadgrove 361	Bald Rocks NP, NSW

*replicates of the species from within the same or nearby populations.

Table S2. *Species, reference and code corresponding to essential oil chemical profiles taken from published data. To see the chemical profiles of these species see cited literature [1,2].*

Affiliation	Collector Reference	Code
P. lasianthos var. lasianthos	D.W. Lawrence 894 (NE)	**LSL-1**
P. lasianthos var. lasianthos	D.W. Lawrence 895 (NE)	**LSL -2**
P. lasianthos var. lasianthos	D.W. Lawrence 896 (NE)	**LSL -3**
P. lasianthos var. subcoriacea	K.L. Wilson 11271 (NSW)	**LSS-1**
P. lasianthos var. subcoriacea	J.R. Nevin 115 (NE)	**LSS -2**
P. lasianthos var. subcoriacea	J.R. Nevin 116 (NE)	**LSS -3**
P. eungella	I.R. Telford 13488a (NE)	**EUA-1**
P. eungella	I.R. Telford 13488b (NE)	**EUA-2**
P. eungella	I.R. Telford 13488 (NE)	**EUA-3**
P. sp. Bald Mountain	N.J. Sadgrove 286 (NE)	**BDM-1**
P. sp. Bald Mountain	N.J. Sadgrove 304 (NE)	**BDM-2**

P. sp. Bald Mountain	N.J. Sadgrove 357 (NE)	**BDM-3**
P. sp. Mount Kaputar	N.J. Sadgrove 276 (NE)	**MTK-1**
P. sp. Point Lookout	N.J. Sadgrove 309 (NE)	**PTL-1**
P. sp. Schofields Gap	N.J. Sadgrove 270 (NE)	**SSG-1**
P. sp. Schofields Gap	N.J. Sadgrove 294 (NE)	**SSG-2**
P. sp. Wollomombi Gorge	N.J. Sadgrove 511 (NE)	**WIG-1**
P. sp. Wollomombi Gorge	N.J. Sadgrove 311 (NE)	**WIG-2**
P. sp. Wollomombi Gorge	N.J. Sadgrove 311a (NE)	**WIG-3**
P. sp. Wollomombi Gorge	N.J. Sadgrove 311b (NE)	**WIG-4**
P. sp. Wollomombi Gorge	N.J. Sadgrove 311c (NE)	**WIG-5**
P. sp. Wollomombi Gorge	N.J. Sadgrove 311d (NE)	**WIG-6**
P. sp. Wollomombi Gorge	N.J. Sadgrove 319 (NE)	**WIG-7**
P. sp. Wollomombi Gorge	N.J. Sadgrove 319a (NE)	**WIG-8**
P. sp. Wollomombi Gorge	N.J. Sadgrove 319b (NE)	**WIG-9**
P. centralis	D185139	**CES-1**

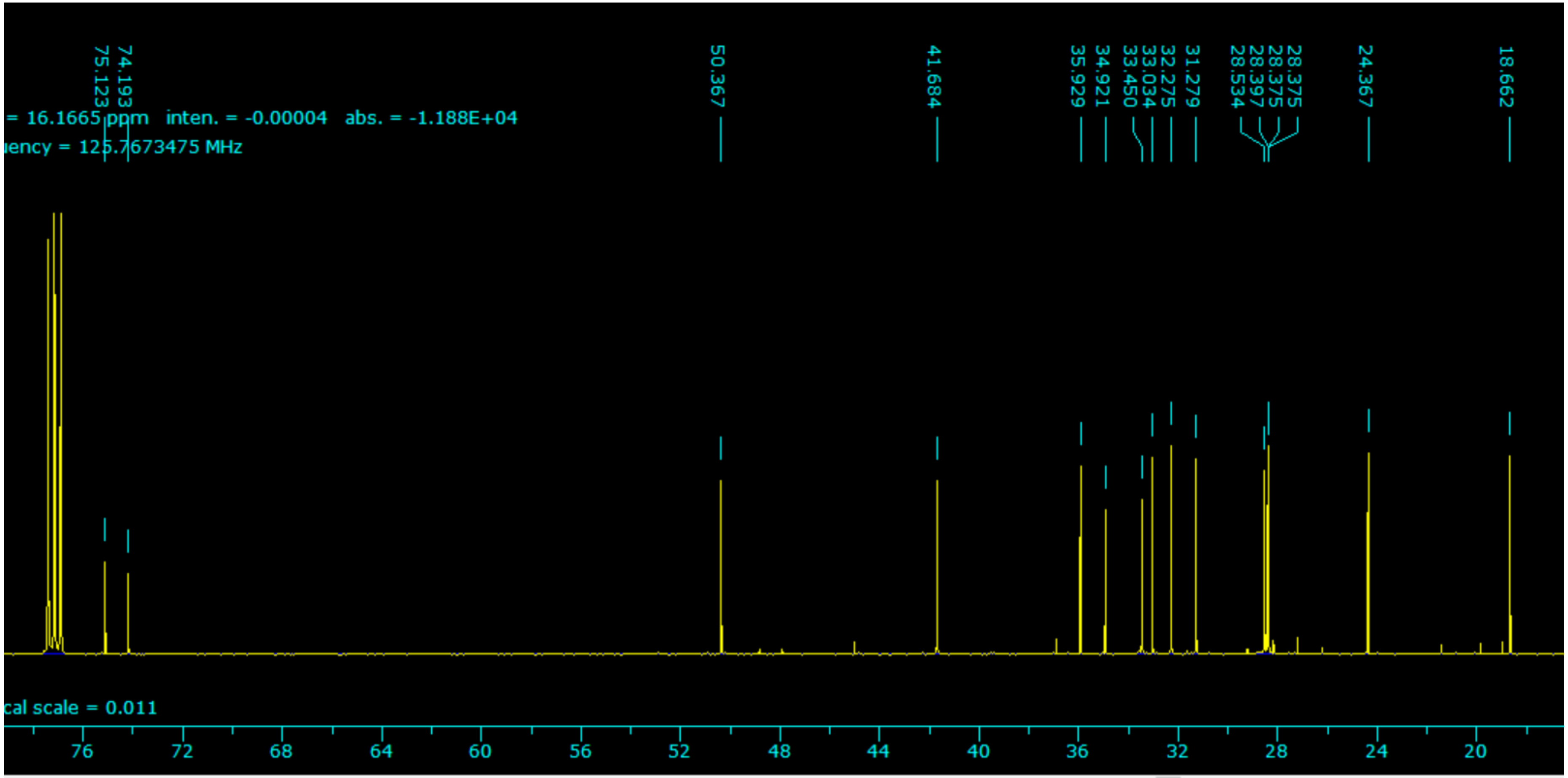

Figure S1 - ^{13}C NMR of kessane isolated from the variagated 'Prostanthera ovalifolia' from a cultivated specimen. Processing software is Spinworks™.

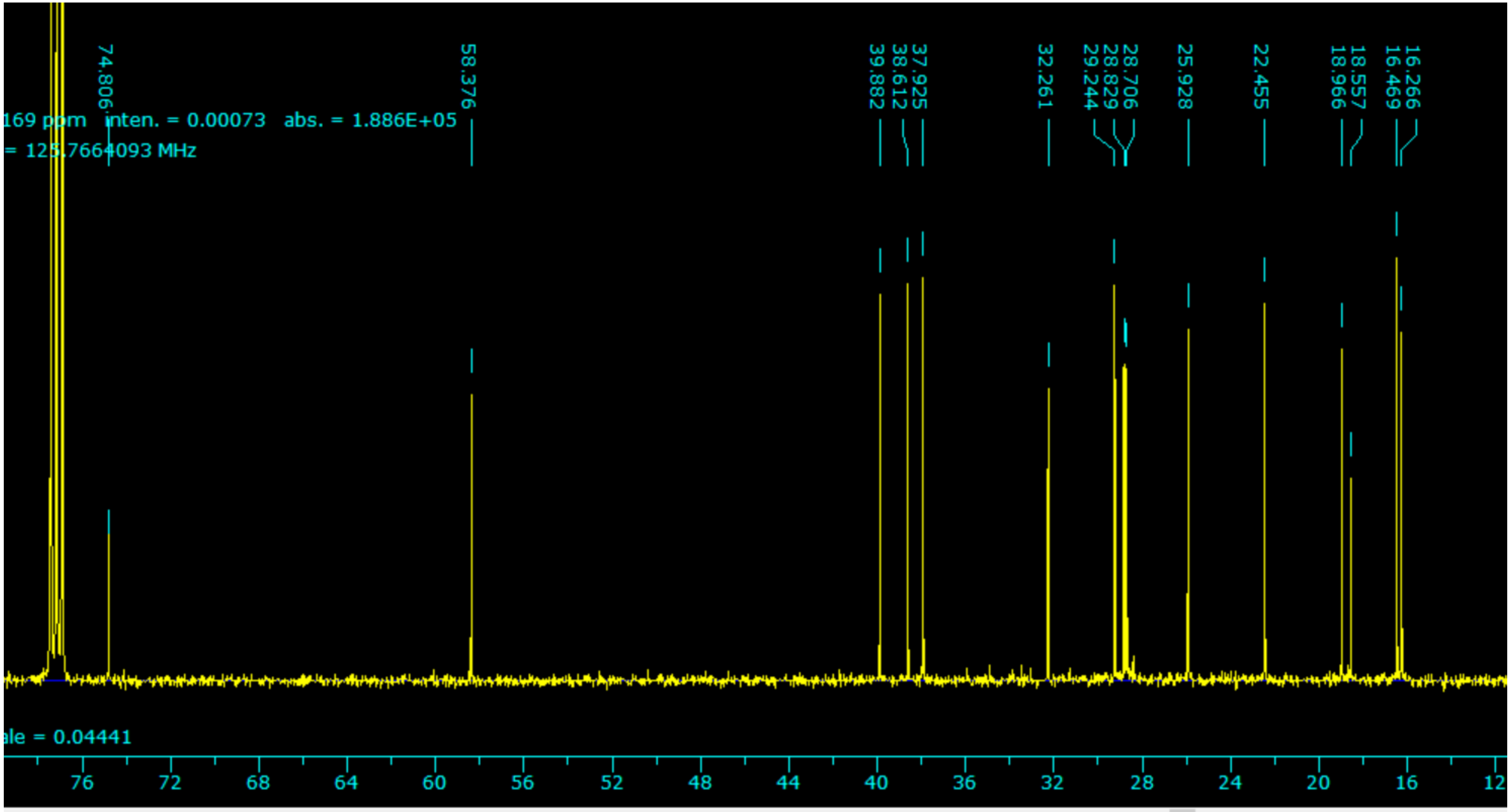

Figure S2 - ^{13}C NMR of pure prostantherol isolated from Prostanthera petraea. Processing software is Spinworks™.

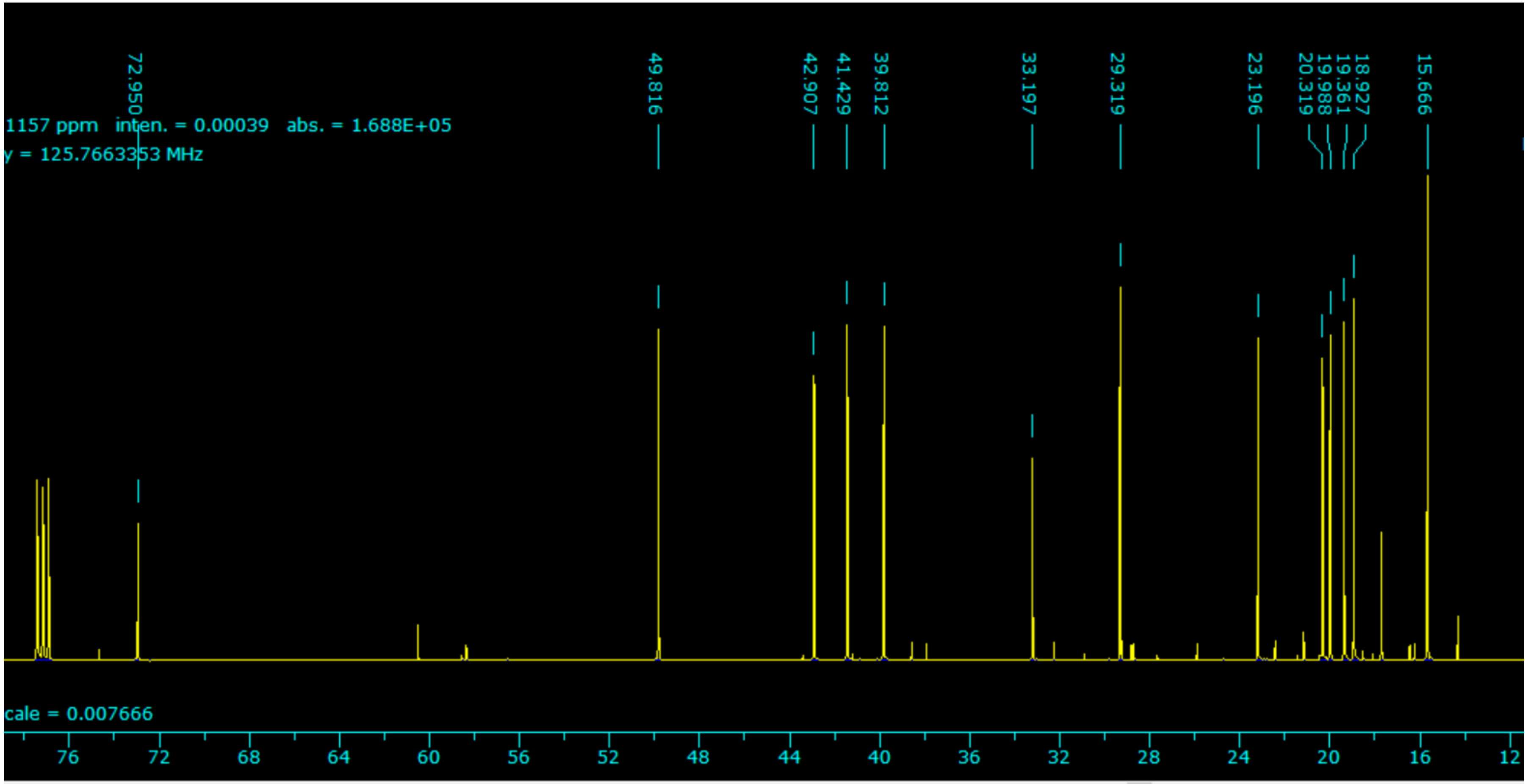

Figure S3 - ^{13}C NMR of near pure maaliol in whole essential oil from Prostanthera lithospermoides. Processing software is Spinworks™.

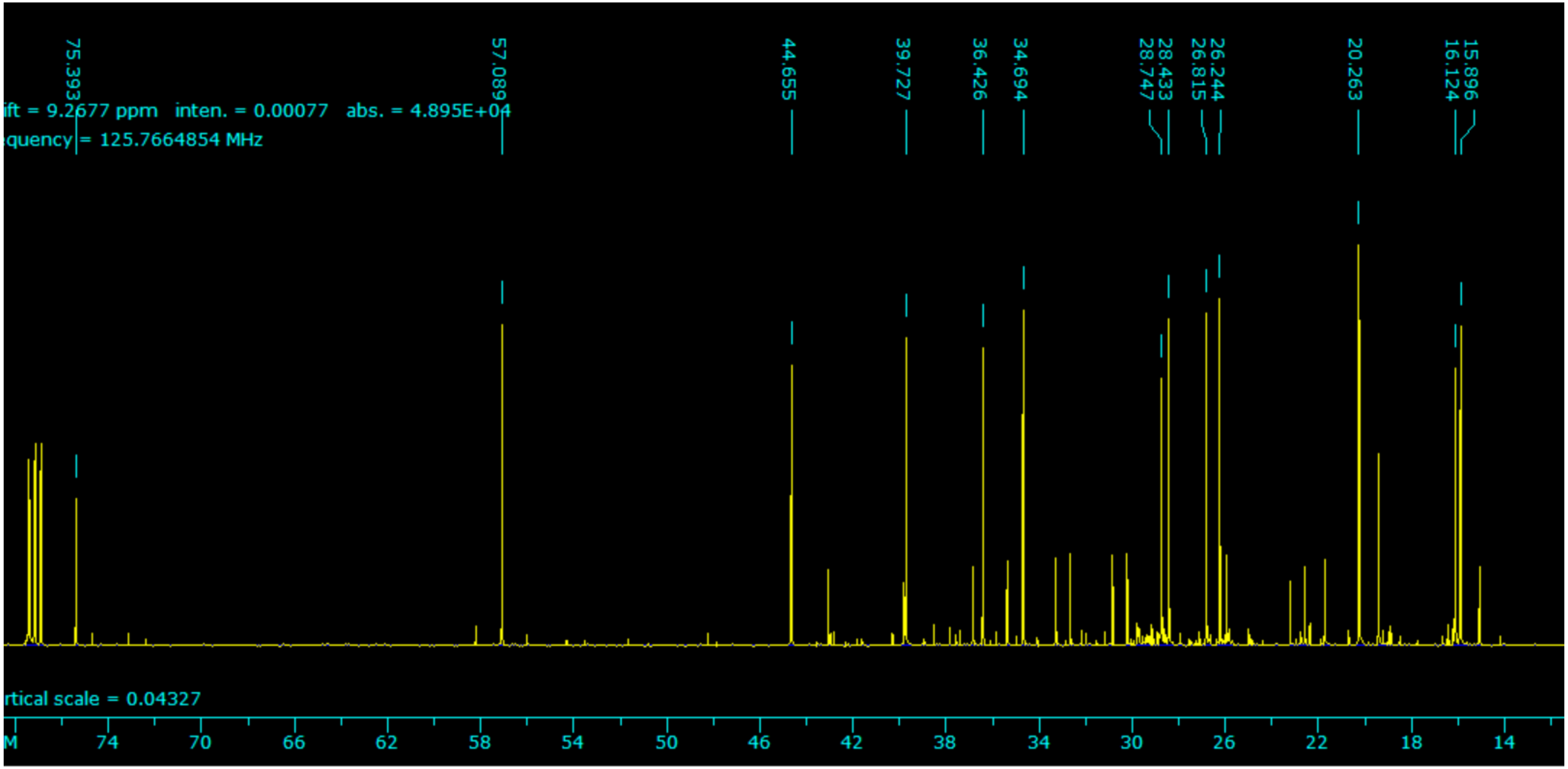

Figure S4 - ^{13}C NMR of globulol in enriched fraction of the essential oil from Prostanthera aspalathoides (collection from condenser by hexane gave globulol and Z-dihydroagarofuran). Processing software is Spinworks™.

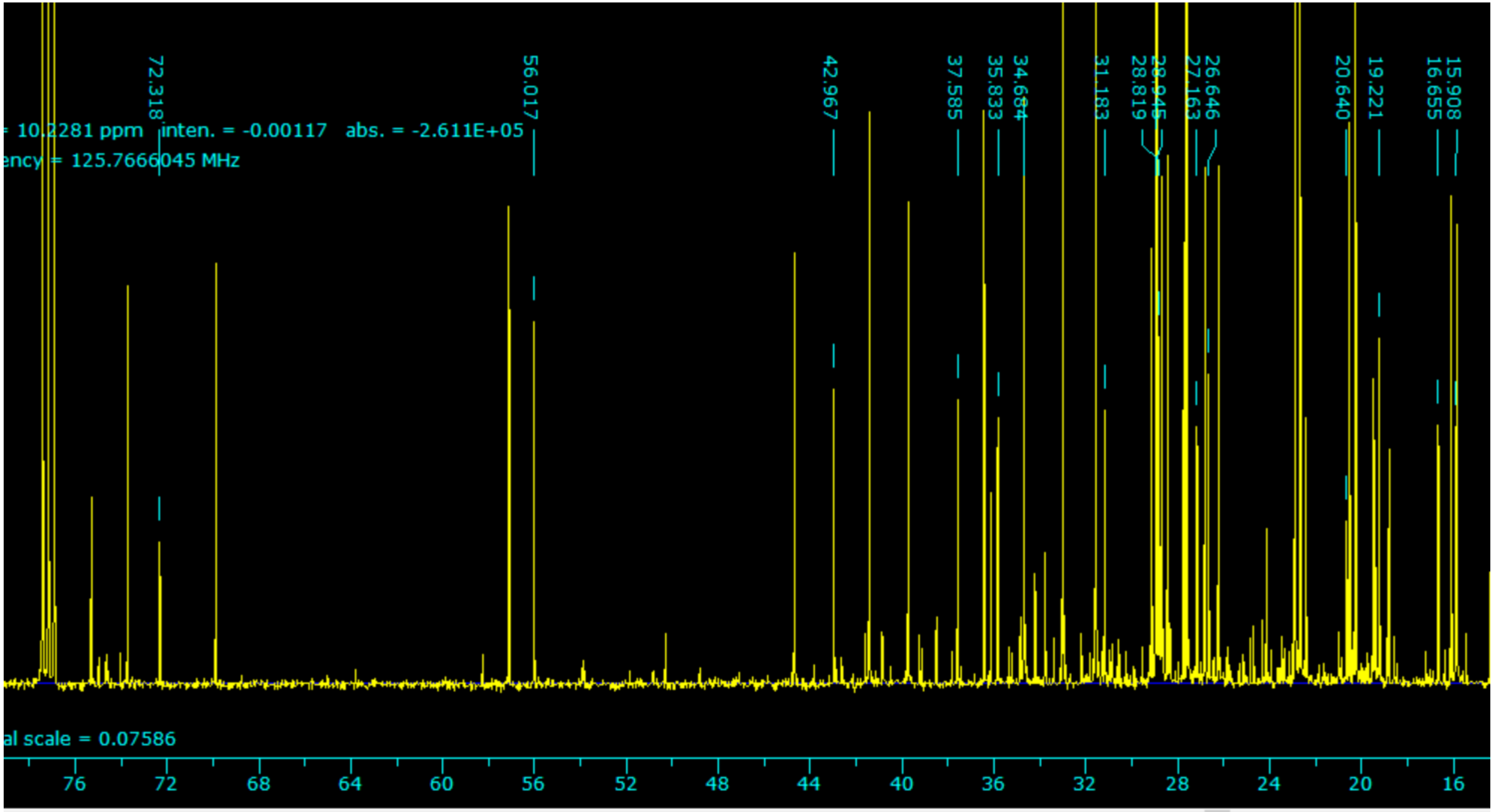

Figure S5 - ^{13}C NMR of epiglobulol as a major component of the whole essential oil of Tasmanian Prostanthera rotundifolia. The selected shifts (ppm) are from the epiglobulol. Processing software is Spinworks™.

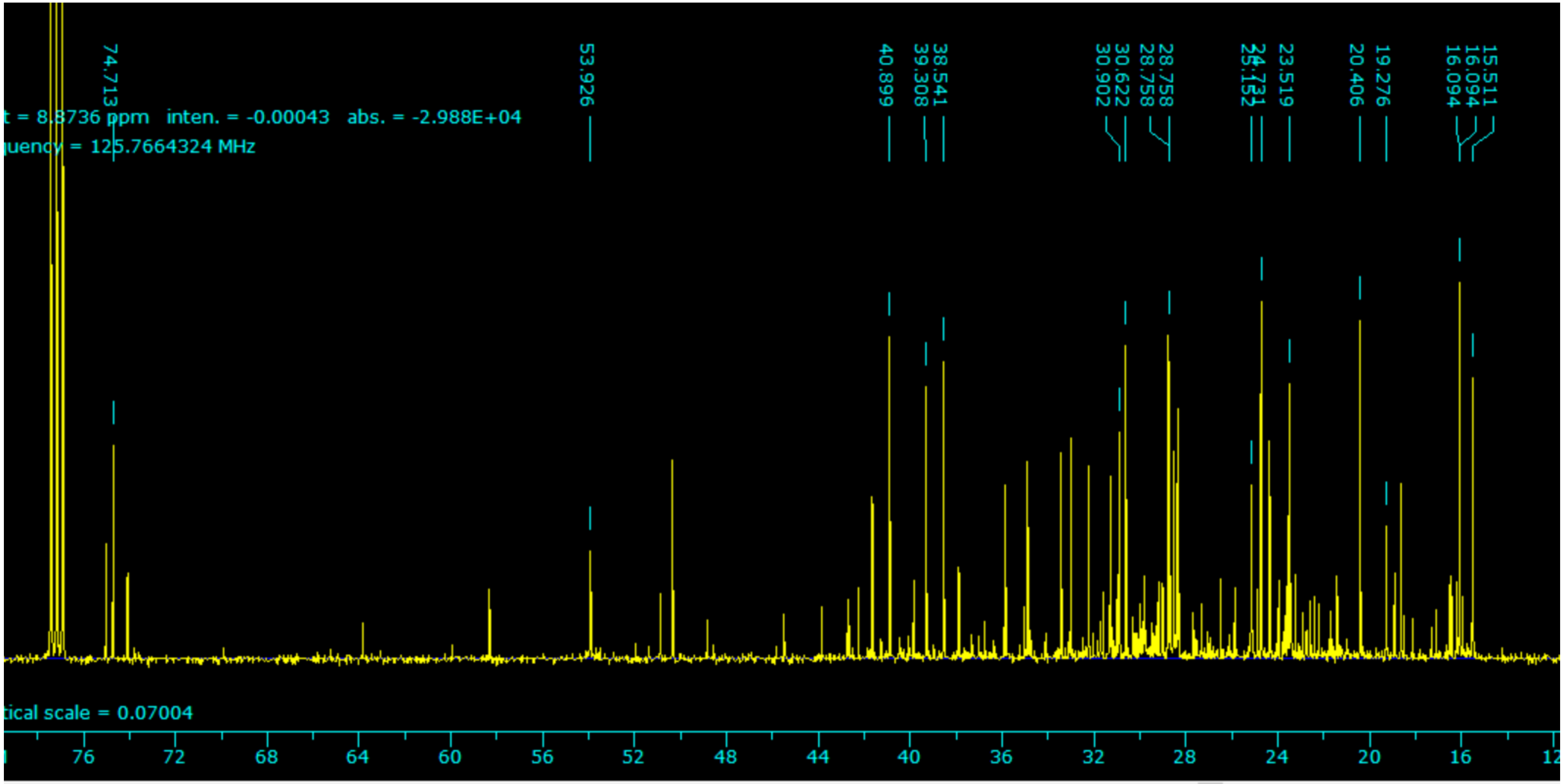

Figure S6 - ^{13}C NMR of ledol in whole essential oil from Tasmanian Prostanthera rotundifolia. The selected shifts (ppm) are for the ledol. Processing software is Spinworks™.

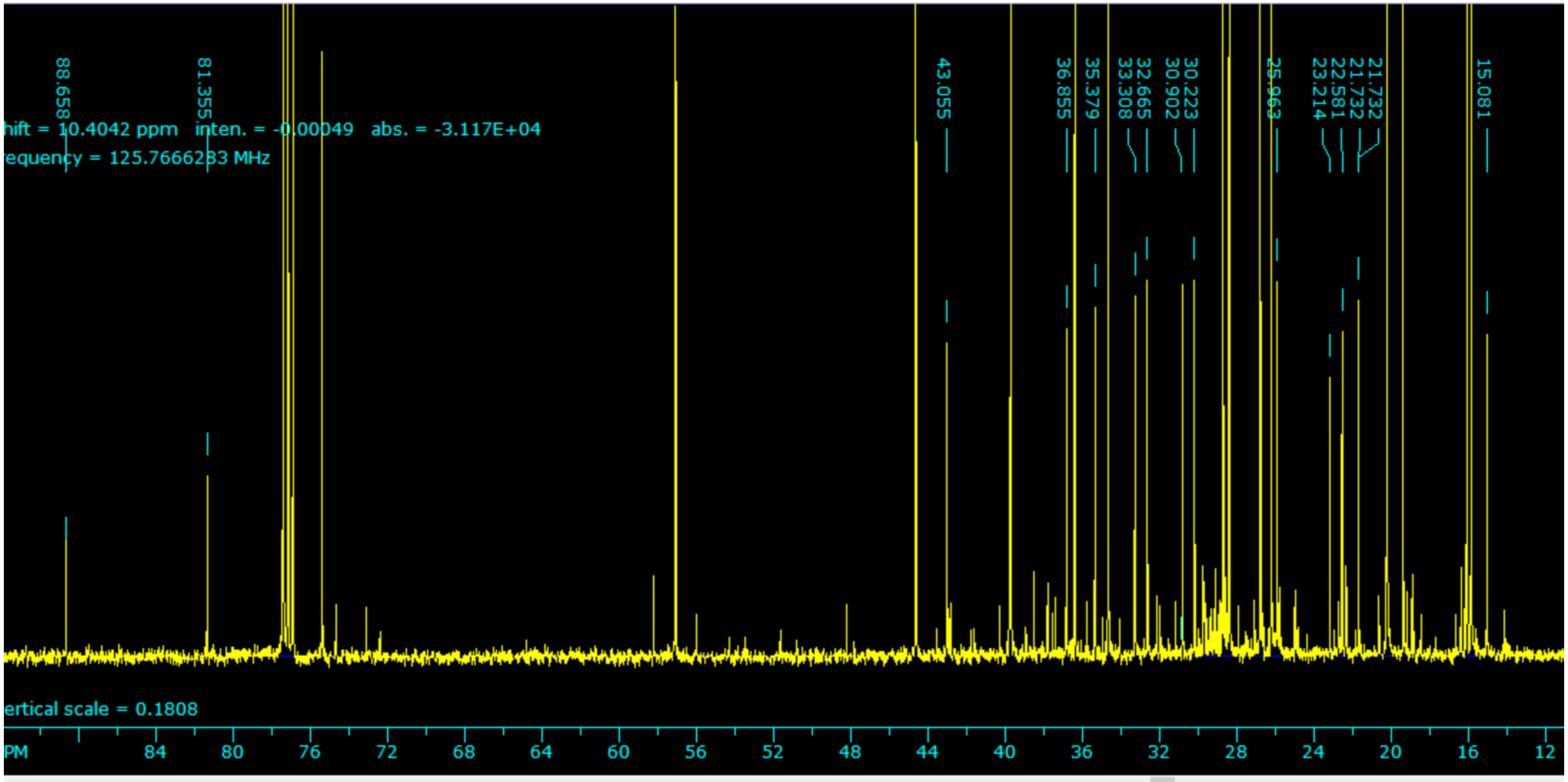

Figure S7 - [13]C of Z-dihydroagarofuran in enriched fraction of essential oil from Prostanthera aspalathoides (collection from the condenser by hexane gave this enriched fraction). Processing software is Spinworks™.

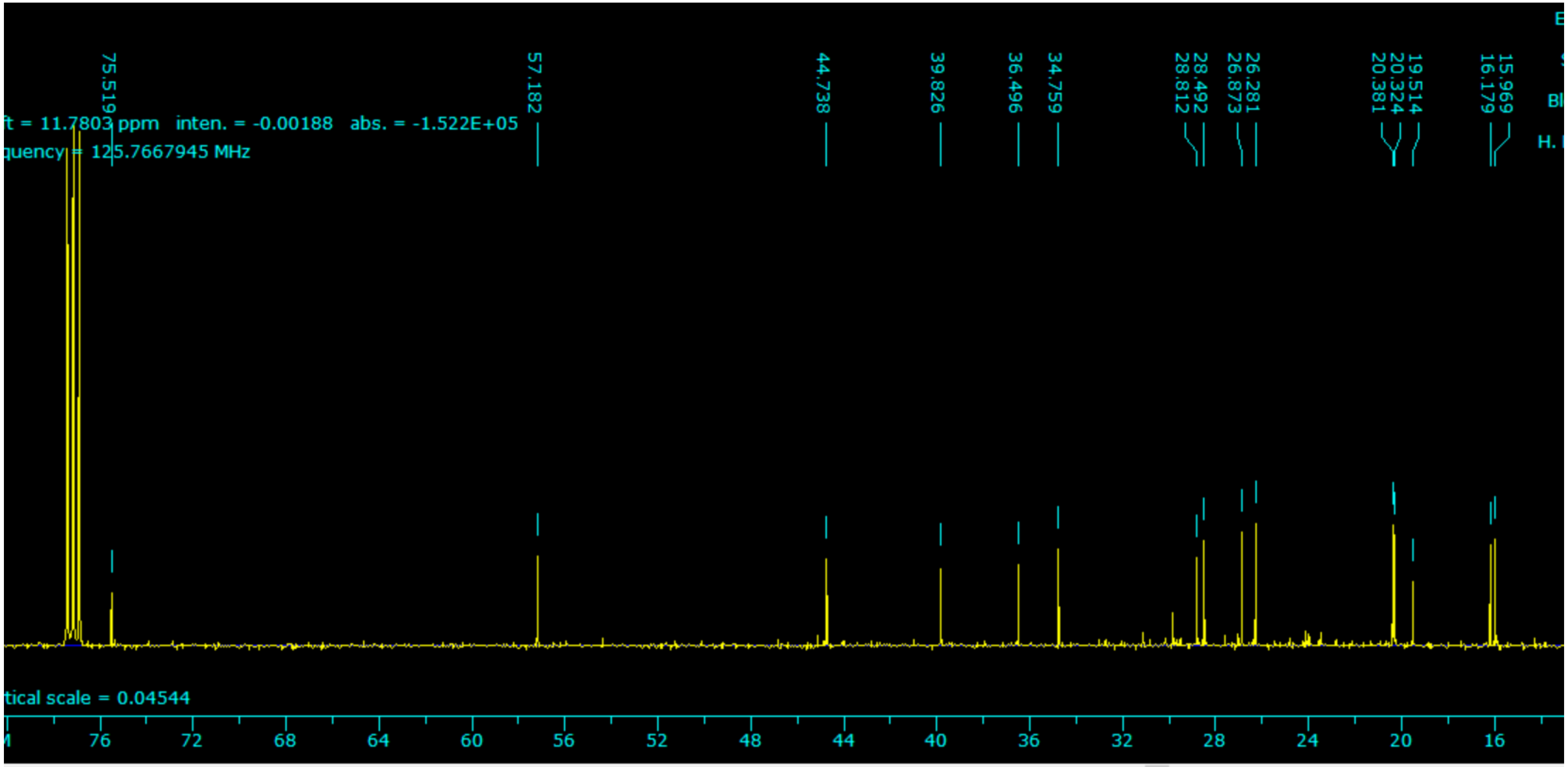

Figure S8 - ^{13}C NMR spectra of globulol isolated from Prostanthera sp. Piliga. Processing software is Spinworks™.

1. Sadgrove, N.J. Comparing essential oils from australia's 'victorian christmas bush' (*prostanthera lasianthos* labill., lamiaceae) to closely allied new species: Phenotypic plasticity and taxonomic variability. *Phytochemistry* **2020**, *176*, 112403.
2. Collins, T.L.; Jones, G.L.; Sadgrove, N. Volatiles from the rare australian desert plant *prostanthera centralis* b.J.Conn (lamiaceae): Chemical composition and antimicrobial activity. *Agriculture* **2014**, *4*, 308-316.
